# *In vivo* lung microbiome alterations from burn pit emissions and/or sand inhalation exposures

**DOI:** 10.3389/fpubh.2025.1693310

**Published:** 2026-04-20

**Authors:** Jeanette S. Frey, Matthew W. Grogg, Andrew J. Hoisington, Brian A. Wong, Karen L. Mumy, Camilla A. Mauzy

**Affiliations:** 1 Henry M. Jackson Foundation for the Advancement of Military Medicine, Wright-Patterson AFB, Dayton, OH, United States; 2 Biotechnology Branch, Air & Space Biosciences Division, Human Effectiveness Directorate, 711th Human Performance Wing, Air Force Research Laboratory, Wright-Patterson AFB, Dayton, OH, United States; 3 Rocky Mountain Mental Illness Research Education and Clinical Center (MIRECC), Rocky Mountain Regional VA Medical Center (RMRVAMC), Aurora, CO, United States; 4 Department of Physical Medicine & Rehabilitation, University of Colorado Anschutz Medical Campus, Aurora, CO, United States; 5 Military and Veteran Microbiome: Consortium for Research and Education, Aurora, CO, United States; 6 Oak Ridge Institute for Science and Education, Oak Ridge, TN, United States; 7 Naval Medical Research Unit Dayton, Wright-Patterson Air Force Base, Dayton, OH, United States

**Keywords:** burn pit exposure, deployment hazards, inhalation exposure, lung microbiome, sand exposure

## Abstract

**Introduction:**

In-theater inhalation exposure to burn pit emissions (BPEs) and sand has been linked to respiratory issues, prompting a study to identify molecular alterations and potential biomarkers related to exposure and outcomes.

**Methods:**

Using a complex *in vivo* exposure scenario to mimic in-theater inhalation exposures, Sprague–Dawley rats were exposed to clean air (Control), BPEs, Sand, or a combination of BPE + Sand via whole-body exposure chambers. After euthanasia, bronchoalveolar lavage fluid was collected at 4 days and 90 days post-exposure, and bacterial amplicon sequence variants were identified using genomic DNA extraction and 16S rRNA gene sequencing.

**Results:**

Both BPE and BPE + Sand exposures significantly altered the lung microbiome, demonstrating increased mean alpha diversity and the highest number of unique ASVs. These changes in the lung microbiome began as early as 4 days post-exposure and continued throughout 90 days post-exposure. BPE and BPE + Sand groups had increased levels of *Bradyrhizobium* and *Methylobacterium* and decreased levels of *Pseudomonas* compared to the Control and Sand groups. The genera most associated with the differences at 4 days post-exposure between the BPE vs. Control and BPE + Sand vs. Control groups were *Corynebacterium*, *Geobacillus*, *Sphingomonas*, and *Streptococcus*. Interestingly, the lung microbiome from the Sand or Control groups was not significantly altered based on alpha or beta diversity and shared the most abundant genera.

**Discussion:**

These data indicate that BPE exposure significantly alter the lung microbiome, whereas sand inhalation exposures alone did not seem to cause significant changes, nor did they provide an additive effect when combined with BPE. While the sub-chronic exposure study design led to more subtle molecular alterations in the lung tissue than expected, BPE exposures resulted in distinct and significant microbiome compositional changes in the lung. The observed population shift provided a signature specific to the type of inhalation exposure. Further efforts could lead to an understanding of the role of individual lung microbiomes in inhalation exposure risks and outcomes.

## 1 Background

Over 2.5 million military personnel have served in Southwest Asia (SWA) since 2002 as part of the Iraq and Afghanistan conflicts (1), and a significant proportion of them have reported respiratory symptoms, predominantly dyspnea (2, 3). In 2010, the Department of Veterans Affairs commissioned the National Academy of Medicine [formerly the Institute of Medicine (IOM)] to convene a committee to examine human health risks from particulate matter (PM) exposure in Iraq and Afghanistan, with a particular focus on exposures from burn pits (4), open areas for burning mixed solid waste (MSW). After review, the IOM recommended a cohort epidemiological study of military personnel and veterans to assess the potential health effects related to burn pit emissions (BPEs) (5). In addition, the IOM recommended an examination of the potential adverse health effects resulting from mixed exposures, including exposure to respirable dust and combustion products.

The potential health effects of the combined inhalation of ambient PM and burn pit emissions are of interest to the Veterans’ Administration. In 2013, a new public law required the Department of Veterans Affairs to establish a registry of veterans who might have been exposed to burn pits in Iraq or Afghanistan. In response, the Airborne Hazards and Open Burn Pit (AH&OBP) Registry was created. The Office of Public Health found that registry participants with documented burn pit exposures were more likely to report respiratory conditions such as chronic obstructive pulmonary disease (COPD), chronic bronchitis, and emphysema (6). Using AH&OBP Registry data and deployment information from the Armed Forces Health Surveillance Center (AFHSC) and the U.S. Department of Defense Manpower Data Center (DMDC), Liu et al. (7) also found that increased days of deployment within 2 miles of burn pits led to a higher incidence of self-reported emphysema, chronic bronchitis, and COPD. Despite these efforts, epidemiological studies have been unable to associate environmental exposures with specific adverse effects, as military personnel may be subjected to a wide variety of uncharacterized levels of airborne particulate matter (PM) and environmental pollutants while deployed.

In spite of the modest association between burn pit exposure and disease (8), it has long been hypothesized that airborne PM, polyaromatic hydrocarbons (PAHs), and volatile organic compounds (VOCs) promote respiratory disease onset (6–9). Studies on general population exposures to air pollution indicate that major air pollution incidents are associated with increases in morbidity and mortality rates (10–12). Concerns regarding human exposure to burn pit emissions (BPE), which likely contain VOCs, PAHs, and PM, prompted the U.S. DoD to characterize the chemical content of ambient air in the vicinity of burn pits at the Balad Air Base in Iraq (9). These emissions contain numerous products from complete and incomplete combustion, including PM, PAHs, and VOCs such as carbon monoxide (CO), sulfur dioxide (SO_2_), and nitrogen dioxide (NO_2_) (10). An additional concern regarding deployment inhalation exposures is the long-term morbidity caused by the inhalation of PM with a particle size distribution of less than 2.5 micrometers (μm) in diameter (PM_2.5_) (11). Studies have shown that death rates increase with higher air pollution concentrations of PM_2.5_ (12, 13). In addition to the PM generated by burn pits, personnel deployed to the Middle East may have been exposed to environmental PM concentrations from respirable dust and sand that exceed the established occupational (Occupational Safety and Health Administration), military (Military Exposure Guidelines for Deployed Military Personnel), and international (World Health Organization) safety standards (14). Major dust storms (>1 mg/m^3^) can occur several times per year and persist for days, with moderate dust events (0.2–1 mg/m^3^) occurring more frequently (15). Depending on locality, 10–40% of SWA sand particles have an aerodynamic diameter of less than 2.5 microns (14, 16) and are therefore capable of reaching the lower lung.

Since the lung is the primary target organ for PM and other emission chemicals, several studies have examined molecular changes in lung tissues initiated by PM, VOC, and PAH inhalation exposures to determine pathway changes that could lead to disease outcomes (17–19). However, very little is known about the lung commensal microbiome and its role in susceptibility to inhaled toxicants. Until recently, the lung was considered a relatively sterile organ, but with the advancement of culture-independent sequencing methods, it has been shown that the normal lung does contain bacteria, with *Firmicutes*, *Bacteroidetes*, and *Proteobacteria* phyla commonly identified among a wide range of populations (20, 21). Studies indicate that subpopulations occur even within the lung (22, 23), with lower bacterial loads in the lower airway versus the upper airway, and possibly in regional commensal populations (24, 25). While the dynamics of the lung microbiome are not entirely understood, recent studies indicate that an individual’s ‘core’ lung microbiota alters after inhalation exposures and the use of drugs (26, 27). In addition, the lung microbiome is modified when affected by acute and chronic diseases (28). A lack of lung microbiome diversity seems to be linked to reduced lung function, a finding seen in smokers with or without chronic obstructive pulmonary disease (COPD) (29), with several studies describing changes in community richness in chronically diseased airways (30, 31). The airway inflammatory status has also been shown to correlate with specific taxonomic features of the airway microbiome (32). Interestingly, some data suggest that specific early environmental exposures to bacteria block the development of asthma and allergies (33–36), indicating that specific lung microbiome populations might be beneficial.

To expand the understanding of the effects of airborne hazard inhalation on deployed military personnel, an *in vivo* study was initiated to investigate the molecular alterations produced by burn pit emissions and/or sand inhalation using known dosages and well-characterized exposure components. The emission exposures used were characteristic of the 2006–2009 burn pit waste stream generated in the Middle East area of operation. Respirable-sized sand particles collected from Camp Victory (Iraq) were also evaluated with and without BPE exposure. Animal exposures, histopathology, inflammatory response, and inhalation toxicology evaluations were performed by the Naval Medical Research Unit Dayton (NAMRU-D) and are reported in-depth elsewhere (37, 38). This report utilized lung lavage samples to investigate the effects of airborne hazard inhalation on the lung microbiota. We hypothesized that exposures to burn pit emissions, sand, or a combination of the two would initiate specific bacterial shifts in the lung microbiome (LMB) and that those observed population shifts could provide a unique biomarker signature specific to the type of inhalation exposure.

## 2 Methods

### 2.1 Animal exposure model

This study was conducted under an IACUC-approved protocol in a facility accredited by AAALACi in accordance with the Guide for the Care and Use of Laboratory Animals (NRC, 2011). Animal studies were performed according to experimental protocols approved by the AFRL Institutional Animal Care and Use Committee (Protocol Number F-WA-2014-0153A).

**Figure 1:**
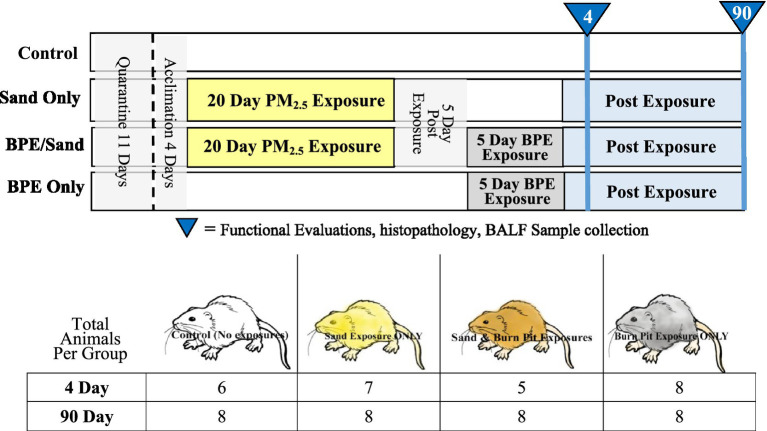
Exposure timeline and sample size. Each test group contained two sets of animals (4-day-old and 90-day-old) that underwent necropsies and BALF collection after euthanasia at the designated time point. BPE = Burn Pit Emission Exposure Group; Sand = Sand Exposure Group; BPE/Sand = Sand plus Burn Pit Emission Exposure Group. PM_2.5_ = Particulate matter with a diameter of 2.5 mm or less.

Male Sprague–Dawley rats (*Rattus norvegicus*) were exposed to clean air (control), sand, burn pit emissions, or a sequential combination of the two (*n* = 8 per exposure group) through inhalation in whole-body exposure chambers. Sand exposures consisted of aerosols of Southwest Asia particulate matter (PM) generated from topsoil collected from Camp Victory, Iraq. The PM aerosol was respirable with a mass median aerodynamic diameter of 2.3 μm and an average mass concentration of 4 mg/m^3^. The rats were exposed to inhalation at a concentration of 5 mg/m^3^ for 20 h/day, 5 days/week, for 20 exposure days over a 4-week period. Burn pit exposures consisted of combustion emissions of municipal solid waste, historically representative of the 2006–2009 waste stream generated in the Middle East area of operation (16.3% plastics, 19.1% wood, 0.2% dunnage, 5% metals, and 59.2% miscellaneous combustible materials) (39). The emission exposure scenario utilized the ambient breeze tunnel (ABT) test facility operated by Battelle (West Jefferson, OH) to simulate open atmospheric conditions and concentrate the smoke plume generated by open burns. The ABT consisted of a large tunnel with a semi-cylindrical cross-section, measuring 6 m high × 6 m wide × 45 m long, made from commercially available materials. Waste was combusted in an open stainless steel pan located at the inlet end of the tunnel. A fan at the outlet end of the tunnel was used to generate airflow through the tunnel to simulate a breeze of up to 5 mph. Sampling and monitoring devices were placed at various points in the tunnel downwind of the source. A variety of instruments were used to characterize the burn plume, including levels of sulfur dioxide (SO_2_), carbon monoxide (CO), carbon dioxide (CO_2_), total hydrocarbons, PAHs, carbonyl compounds, VOCs, dioxins, particle mass, size distribution, and composition [for full details, see Wong et al. (37)]. Gaseous and particulate pollutant species from the plume were transferred to whole-body inhalation chambers located within an ABT-adjacent climate-controlled mobile laboratory. The rats were exposed to an average concentration of 0.5 mg/m^3^ for 6 h/day for 5 consecutive days. The burn pit aerosol had an MMAD of 0.4 μm and a concentration of 0.8 mg/m^3^. CO_2_ and CO in the burn pit exposure averaged 666 ppm and 9.6 ppm, respectively. Recovery from toxic effects was observed at 4 and 90 days post-exposure (Figure 1). In addition, functional evaluations and lung histopathology were performed [described in Wong et al. (37)].

### 2.2 Bronchoalveolar lavage fluid collection

Rats were euthanized by deep anesthetization with ketamine and xylazine, followed by exsanguination via transection of the abdominal aorta prior to necropsy. Bronchoalveolar lavage was performed by infusing and withdrawing 3 × 3 mL of ice-cold, sterile 1X phosphate-buffered saline (PBS) from the left lung. Bronchoalveolar lavage fluid (BALF) was collected in a chilled 15 mL conical centrifuge tube. Approximately 7–8 mL of BALF was recovered. BALF was centrifuged for 10 min at 200 × g and 4 °C. BALF supernatant was stored at −80 °C.

### 2.3 DNA extraction

A total of 2 mL of BALF was centrifuged at 110,000 × g for 80 min at 4 °C. BALF pellets were extracted using a NucleoSpin Tissue Kit (Macherey-Nagel, Cat. No. 740952), according to the manufacturer’s instructions (Genomic DNA from Tissue—User Manual, January 2017/Rev. 17). BALF pellets were resuspended in 180 uL Buffer T1 and 25 uL Proteinase K. Samples were incubated at 56 °C and 600 rpm (Eppendorf, ThermoMixer C) overnight. After the addition of 200 uL Buffer B3, the samples were incubated at 70 °C for 10 min. A total of 210 uL of 100% ethanol was added, and the samples were vortexed vigorously. Then, the samples were applied to NucleoSpin^®^ Tissue Columns and centrifuged for 1 min at 11,000 × g. Columns were washed with 500 uL Buffer BW for 1 min at 11,000 × g, followed by 600 uL Buffer B5 for 1 min at 11,000 × g. Genomic DNA (gDNA) was eluted with 60 uL Buffer BE (70 °C) for 1 min at 11,000 × g. The eluted gDNA was stored at −80 °C.

### 2.4 16S rRNA gene sequencing

The bacterial 16S rRNA gene hypervariable regions were amplified using an Ion 16S Metagenomics Kit (Thermo Fisher Scientific Cat. No. A26216) according to the manufacturer’s instructions (Ion 16S Metagenomics Kit; Pub. No. MAN0010799 Revision C.0) with two protocol changes: first, the DNA input was increased to 12 uL and second, the number of amplification cycles increased to 30. PCR products were run on a 2% agarose gel to confirm amplification prior to purification with Agencourt AMPure XP beads (Thermo Fisher Scientific Cat. No. A26216). Amplicons were quantified using the Agilent 4,200 TapeStation System (Cat. No. G2991AA), and D1000 ScreenTape (Cat. No. 5067-5582) and reagents (Cat. No. 5067-5583) according to the manufacturer’s instructions (Agilent D1000 ScreenTape Assay Quick Guide for 4200 TapeStation System, G2991-90030 Rev. B Edition 02/2017). Amplicons were end-repaired using an Ion Plus Fragment Library Kit (Thermo Fisher Scientific Cat. No. 4471252) and 50 ng of amplicons in a 79 uL volume. Amplicons were barcoded using an Ion Xpress Barcode Adaptor 1–96 Kit (Thermo Fisher Scientific Cat. No. 4474517). The adaptor-ligated and nick-repaired DNA was purified using Agencourt AMPure XP beads and amplified using an Ion Plus Fragment Library Kit. The amplified library was again purified using Agencourt AMPure XP beads and quantified using Agilent High Sensitivity D1000 ScreenTape (Cat. No. 5067-5584) and reagents (Cat. No. 5067-5585) according to the manufacturer’s instructions (Agilent High Sensitivity D1000 ScreenTape Assay Quick Guide for 4200 TapeStation System G2991-90130 Rev. D Edition 02/2017). Each library was diluted to 60 pM. In the six samples, the amount and quality of the library were insufficient for template preparation. Therefore, these samples were excluded from sequencing, and the sample size was reduced for these exposure groups (Figure 1). A total of 58 samples were included in the analysis.

Diluted libraries were pooled for template preparation and chip loading using an Ion Chef System (Thermo Fisher Scientific, Cat. No. 4484177). The Ion Chef was set up according to the manufacturer’s instructions (Ion 520 and 530 Kit-Chef User Guide, Pub. No. MAN0010846) using Ion 520 and 530 Kit-Chef (Thermo Fisher Scientific Cat. No. A27755) and Ion 530 Chip Kit (Thermo Fisher Scientific Cat. No. A27764). Sequencing was performed using an Ion S5 System (Thermo Fisher Scientific Cat. No. A27212) using Ion 520 and 530 Kit-Chef and Ion 530 Chip Kits, according to the manufacturer’s protocol. Base calling and demultiplexing were performed using Torrent Suite software, v 5.0, with default parameters.

### 2.5 16S rRNA gene sequence processing

Single-end demultiplexed sequences were uploaded into the QIIME 2 software platform (version 2019–10) (40) for quality control and taxonomic assignments. A total of 37,655,184 sequences in the 58 samples were processed using the default values of the DADA2 denoise pipeline, with the exception of “trim-left” set to 15 and “trunc-len” set to 250. Due to the variable primers in the Ion Torrent system, the optimal taxonomic assignment was found using Greengenes full reference 16S rRNA gene sequences (version 13_8) (41) and the BLAST+ classifier (42). Sequences assigned to mitochondria, chloroplasts, or non-bacterial phyla were removed. After quality control, the dataset included 17,311,149 sequences (192,346 ± 92,303 sequences/sample; mean ± one standard deviation).

### 2.6 Statistical analysis

Samples were rarefied to 40,000 sequences per sample, and all samples were stored for analysis. Statistical analysis was conducted using QIIME 2 (version 2019–10) and R (version 3.6.0) with the phyloseq package (version 1.28.0) (43). DESeq2 (version 1.26.0) was conducted in R (44). Alpha diversity metrics were assessed using the Shannon Diversity Index and the beta diversity metric, weighted UniFrac. Statistical tests for comparisons between groups for alpha and beta diversity were conducted using Wilcoxon signed-rank tests (Wilcoxon Test). Taxonomic changes at the genus level were identified for each exposure from day 4 to day 90 through DeSeq2 using the Wald test and an alpha level set at 0.01.

## 3 Results

### 3.1 Characterization of lung responses to toxicant challenge

Although not included in this report, cytokine analyses and histopathology were conducted on tissues and bodily fluids obtained from this study. IFN-γ, IL-1β, IL-4, IL-5, IL-6, IL-10, IL-13, KC/GRO, and TNF-α levels were evaluated in BALF using ELISA or electrochemiluminescence-based assays (38). In summary, significant increases in TNF-α were seen in the Sand and BPE + Sand groups compared to controls. This elevation continued for up to 90 days post-exposure in the BPE + Sand group. No statistically significant changes were observed in any exposure group for any of the other cytokines evaluated.

Histopathology of the lung tissue revealed minimal-to-mild changes across all exposure groups. Foreign matter was found in tissues from the Sand and BPE + Sand groups. Neutrophilic infiltration was seen in tissues from all exposure groups but was found more frequently in the BPE + Sand group. Interestingly, low levels of hemorrhage were observed in the BPE and BPE + Sand groups, particularly in the 4-day exposure samples (37).

### 3.2 Beta diversity

**Figure 2:**
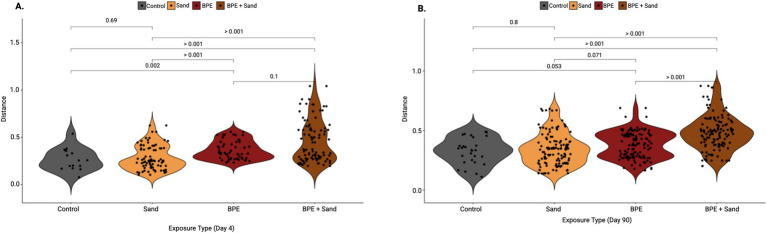
Weighted UniFrac distances as compared to Control, separated by either 4-day post-exposure **(A)** or 90-day post-exposure **(B)**. Pairwise statistics (*p*-values) for each set of samples were obtained using the Wilcoxon test. BPE = Burn Pit Emission Exposure Group; Sand = Sand Exposure Group; BPE + Sand = Sand plus Burn Pit Emission Exposure Group.

To investigate the impact of exposure on the lung microbiome, two time points were analyzed individually for each of the three exposure groups and the control group (Figure 1). At 4 days post-exposure, the beta diversity in weighted UniFrac measures was significantly different in-group comparisons of Control vs. BPE, Control vs. BPE + Sand, Sand vs. BPE, and Sand vs. BPE + Sand (Figure 2A). At 90 days post-exposure, weighted UniFrac beta diversity was significantly different in comparisons of Control vs. BPE + Sand, Sand vs. BPE, Sand vs. BPE + Sand, and BPE vs. BPE + Sand (Figure 2B). There were no significant weighted UniFrac beta diversity differences seen at 4 days or 90 days post-exposure between the Control and Sand groups. Exposure to BPE with and without sand exposure significantly altered the lung microbiome versus Sand or Control groups. The only exception was the nearly significance observed at Day 90 with Control vs. BPE at *p* = 0.053).

**Figure 3:**
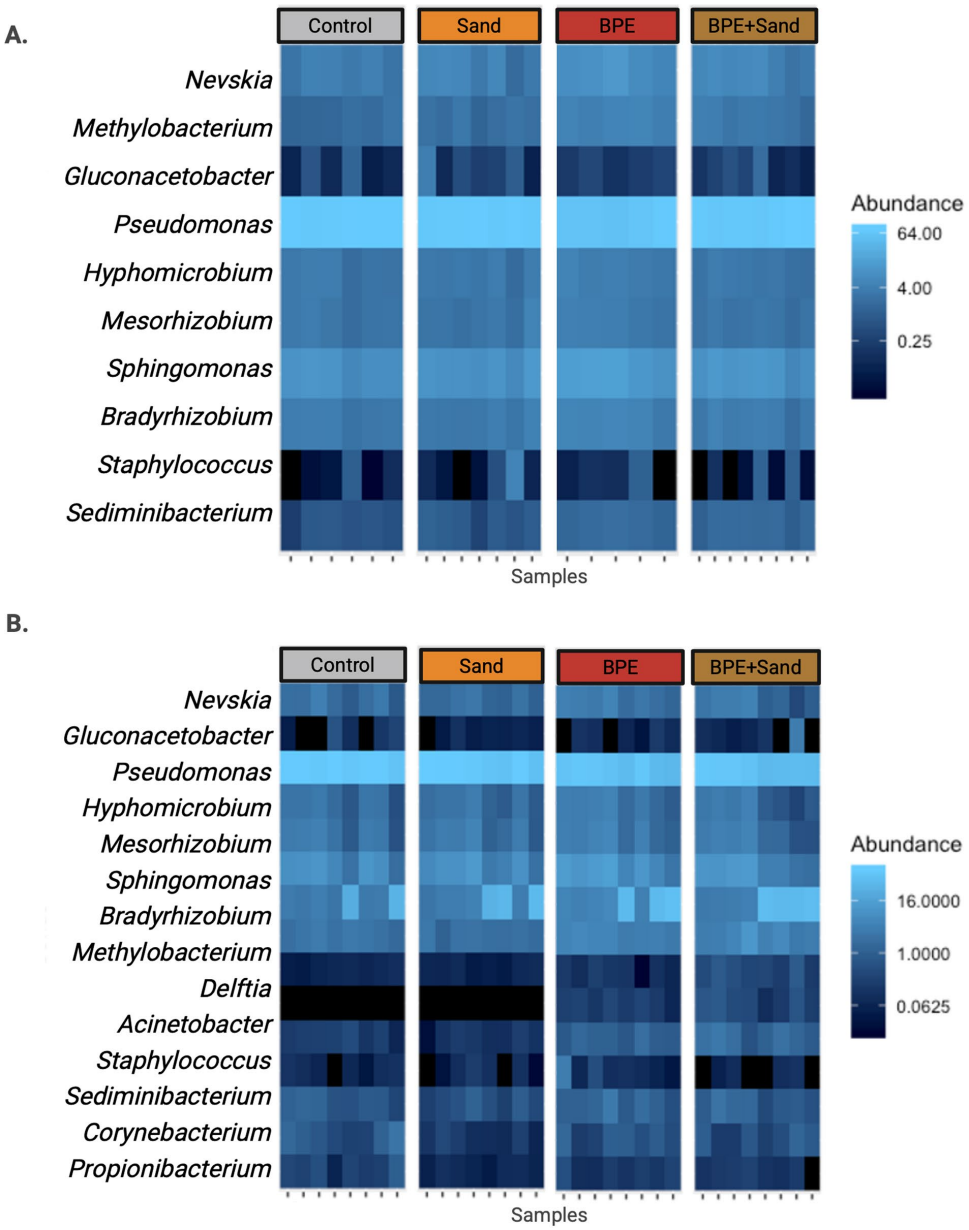
Top 100 most abundant ASVs, separated by either 4-day post-exposure **(A)** or 90-day post-exposure **(B)**, sorted by phyla. BPE = Burn Pit Emission Exposure Group; Sand = Sand Exposure Group; BPE + Sand = Sand plus Burn Pit Emission Exposure Group.

Analyzing taxonomy at the genus level, the most abundant taxa in all groups were similar at 4-day post-exposure (Figure 3A), with the top four most abundant genera being *Pseudomonas*, *Bradyrhizobium*, *Nevskia*, and *Sphingomonas*. Eight of the 10 most abundant genera belonged to the *Proteobacteria* phylum. However, at 90-day post-exposure, genus-level differences were observed (Figure 3B). Notably, the BPE and BPE + Sand groups had increased levels of *Bradyrhizobium and Methylobacterium* and decreased levels of *Pseudomonas* compared to either the Control or Sand exposure groups.

Examination of the top 100 amplicon sequence variants (ASVs) showed many similarities in community structure at 4- and 90-day post-exposure, and all 10 genera from 4-day post-exposure were captured at the later 90-day time point. However, differences were also observed, as *Comamonas*, *Delftia*, and *Gluconacetobacter* were only seen at 90-day post-exposure. Additional taxonomic diversity was observed at 90 days vs. 4 days post-exposure, with 5 of the 15 most abundant taxa belonging to non-Proteobacteria phyla.

### 3.3 Alpha diversity

**Figure 4:**
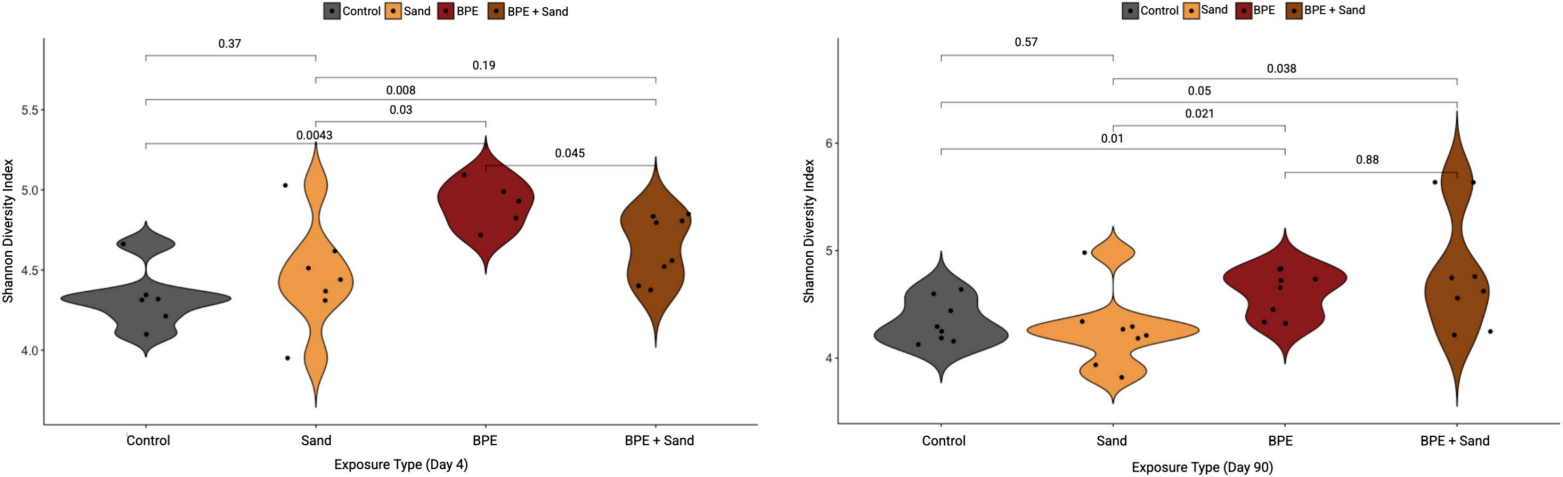
Shannon diversity index by type of exposure, separated by either 4-day post-exposure **(A)** or 90-day post-exposure **(B)**. Pairwise statistics (*p*-value) for each set of samples from the Wilcoxon test. BPE = Burn Pit Emission Exposure Group; Sand = Sand Exposure Group; BPE + Sand = Sand plus Burn Pit Emission Exposure Group.

At 4-day post-exposure, the alpha diversity, measured through the Shannon diversity index, was significantly different in comparisons of Control vs. BPE, Control vs. BPE + Sand, BPE vs. BPE + Sand, and Sand vs. BPE (Figure 4A). No significant differences were observed between Control vs. Sand and Sand vs. BPE + Sand. At 90-day post-exposure, the alpha diversity was significantly different in comparisons of Control vs. BPE, Control vs. BPE + Sand, Sand vs. BPE, and Sand vs. BPE + Sand (Figure 4B). No significant differences were observed between Control vs. Sand and BPE vs. BPE + Sand. At both 4-day and 90-day post-exposures, the BPE and BPE + Sand groups had higher Shannon diversity indices than the Sand or Control groups. In addition, alpha diversity significance through the Shannon diversity index revealed a similar pattern as beta diversity, namely BPE and BPE + S, and groups had similar values that were significantly different from either Control or Sand. Overall, the highest unique ASVs occurred in the BPE exposure group, followed by the BPE + Sand, Control, and Sand groups (Supplementary Figure 4).

**Figure 5:**
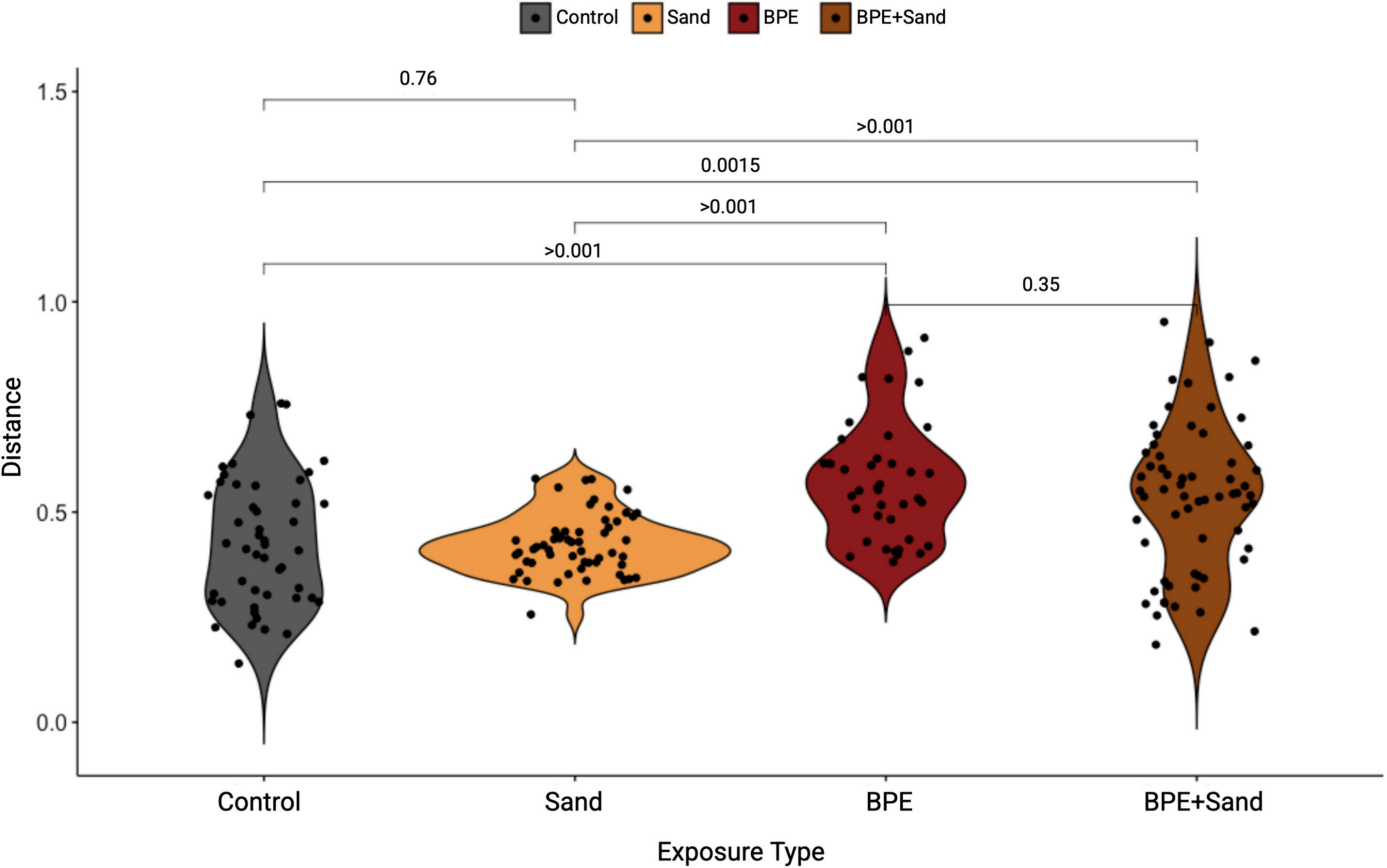
Weighted UniFrac distances for each exposure type, comparing beta diversity between 4 days and 90 days after exposure. BPE = Burn Pit Emission Exposure Group; Sand = Sand Exposure Group; BPE + Sand = Sand plus Burn Pit Emission Exposure Group.

When comparing microbiomes within an exposure type (4 days vs. 90 days), both the Control and the ‘BPE + Sand’ groups had high variability (Figure 5). Microbiome community shifts, as defined by a higher weighted UniFrac distance, were highest between 4 days and 90 days in the BPE and the ‘BPE + Sand’ exposure groups, indicating a defined response specific to BPE. Conversely, sand exposures seemed to initiate only minimal shifts in the lung community between 4 days and 90 days. To examine the effects of time on the community, the top 100 ASVs for each exposure group were determined (Supplementary Figures 1A–D). Within the Control group, several genera appeared to increase from 4 to 90 days, notably *Propionibacterium* and *Corynebacterium*. In the Sand group, *Gluconacetobacter* and *Staphylococcus* showed a decreasing trend from 4 to 90 days. The BPE group showed a trending increase in *Bradyrhizobium*, while the ‘BPE + Sand’ group demonstrated increases in *Bradyrhizobium*, *Delftia*, and *Comamonas*.

**Figure 6:**
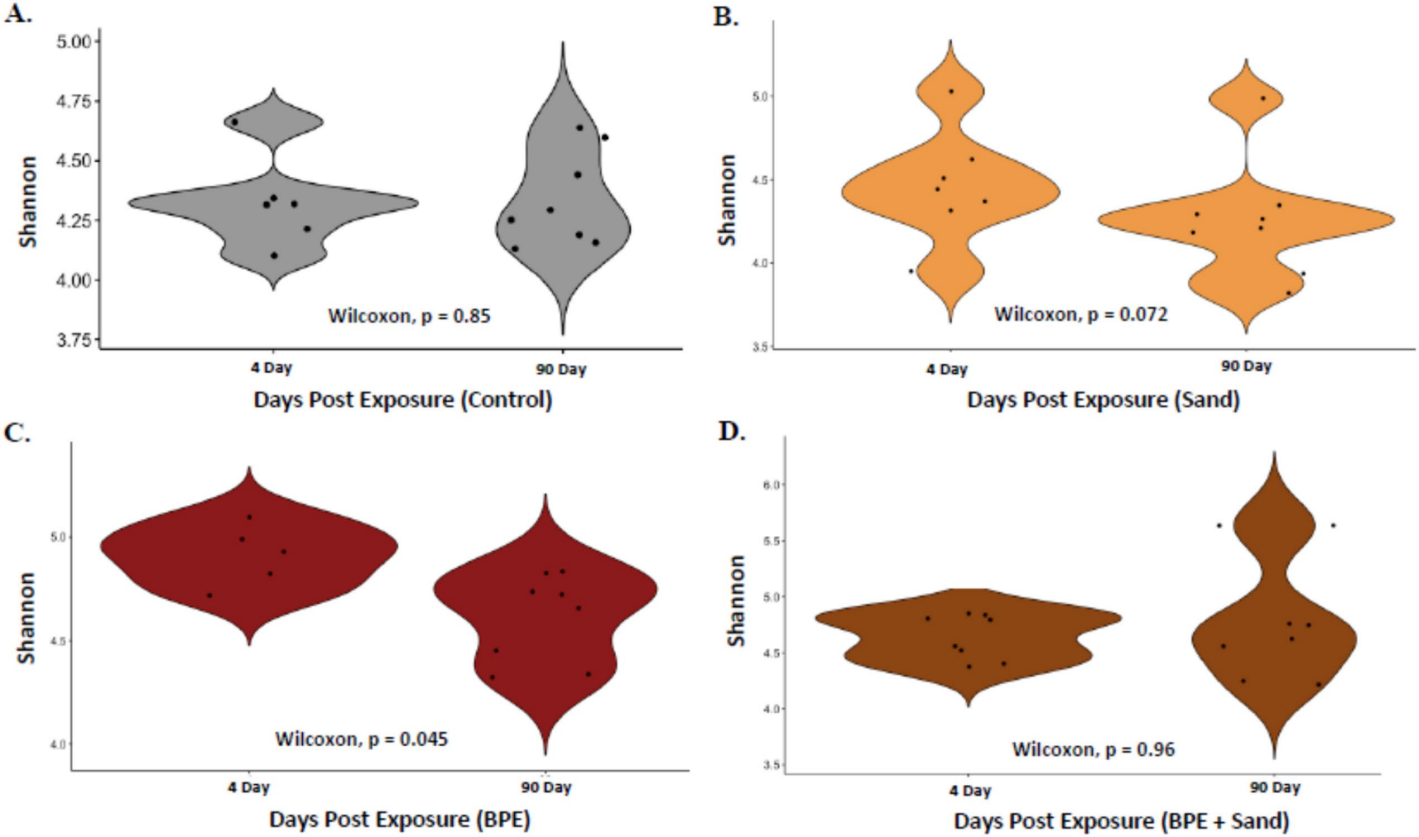
Shannon diversity index of LMB by post-exposure days, separated by **(A)** Control, **(B)** Sand, **(C)** BPE, and **(D)** ‘BPE + Sand’ groups. Pairwise statistics (*p*-value) for each set of samples from the Wilcoxon test. BPE = Burn Pit Emission Exposure Group; Sand = Sand Exposure Group; BPE + Sand = Sand plus Burn Pit Emission Exposure Group.

As expected, in the Control group, no significant differences were observed in alpha diversity between the 4-day post-exposure group and the 90-day post-exposure groups (Figure 6A). Additionally, there were no significant differences observed between the two time points in the Sand or the ‘BPE + Sand’ exposure groups (Figures 6B,D). In the BPE group, however, a higher Shannon diversity index was observed at 4-day post-exposure than at 90-day post-exposure (Figure 6C). Overall, samples collected at 4-day post-exposure had higher unique ASVs compared to 90-day post-exposure (Supplementary Figure 5).

This study analyzed the taxonomic results that caused shifts in the microbiome as time from exposure increased through DESeq2. To account for microbiome shifts not due to exposure, taxonomy that had the same ASV results as the Control group was eliminated from future consideration for the exposure analysis. In total, 170 ASVs were identified as significantly altered from day 4 to day 90 post-exposure (13 Control, 9 Sand, 51 ‘BPE + Sand’, and 97 BPE), representing 59 different genera (see Supplementary Table 1 for full results). A summary of the results of the selected genera is shown in Table 1. Generally, burn pit emission exposures in either the BPE or ‘BPE + Sand’ groups initiated greater changes in the LMB after exposure.

**Table 1:** Select genus changes from 4 days to 90 days post-exposure through DESeq2 analysis in burn pit emissions (BPE) or sand exposure groups.

Genus	Exposure group	# ASV ↑	# ASV ↓
*Bacteroides*	BPE	5	–
*Bradyrhizobium*	BPE	1	–
	BPE + Sand	3	–
*Lactobacillus*	BPE	–	2
	BPE + Sand	2	–
	Sand	3	–
*Mycobacterium*	BPE	–	1
	BPE + Sand	7	–
*Prevotella*	BPE	–	1
	BPE + Sand	2	–
*Salmonella*	Control	–	4
*Sphingomonas*	BPE	–	12
*Staphylococcus*	BPE	1	6
	BPE + Sand	3	3
	Sand	–	1

False discovery was controlled through the Benjamini–Hochberg procedure. Full details are available in Supplementary Table 1. BPE = Burn Pit Emission Exposure Group; Sand = Sand Exposure Group; Burn + Sand = Sand plus Burn Pit Emission Exposure Group.

## 4 Discussion

Active duty personnel have reported being sickened by smoke emitted from burn pits, both from occupational and incidental exposures (45, 46). In addition, there have been concerns that these exposures, when combined with sand inhalation, may exacerbate the development of lung diseases (47). This study provides a novel examination of the effects of burn pit emissions and sand exposures on the resident lung microbiome. Given the known involvement of the lung microbiota in airway disease and inflammation (48), this initial examination of airborne hazard exposure modulation of the lung microbiome may provide insights into possible new mechanisms by which the commensal lung microbiome could contribute to disease initiation and how it may play a role in individualized risk.

### 4.1 Taxonomic overview

Studies on the lung microbiome are not as numerous as those on other niches, such as the gut, skin, or oral microbiome. The healthy human lung microbiome consists of microbes from the phyla *Firmicutes*, *Bacteroidetes*, *Actinobacteria*, and *Proteobacteria* (20, 49). Common genera observed in the human lung include *Haemophilus*, *Neisseria*, *Prevotella*, *Veillonella*, *Staphylococcus*, and *Streptococcus* (49). This study identified *Proteobacteria* as dominant across all groups compared to healthy human lungs, which is not uncommon in murine studies (50). In a related study, Li et al. (51) also used a rat model and observed *Proteobacteria* at a relative abundance of 75.5%, which is lower than the mean 90.3% observed in our study. While higher abundance of *Proteobacteria* has been associated with disease states in the lungs (49, 52, 53), our data indicated that all exposure groups had relatively lower abundances of *Proteobacteria* compared the Control group. As these were not germ-free animals, it is possible that the rats used in the study all started with a high relative abundance of *Proteobacteria* or acquired it via the environment (e.g., through water, bedding, or food) that might have contributed to the observed levels.

### 4.2 Burn pit emission exposures alter the lung microbiome

Animals exposed to BPEs demonstrated significant alterations in the lung microbiome (Figure 1). Interestingly, exposure to BPEs *increased* rather than decreased the mean alpha diversity, an observation seen in patients with burn inhalation injury (54) and COPD (29) patients. Reports on the impact of negative health conditions on LMB alpha diversity are inconsistent, as Pragman et al. (55) reported increased diversity with more severe COPD. However, in their study, it is likely that LMB changes are also influenced by the severity, duration, and age of onset of the disease—parameters well controlled in this study. Beta diversity seen in the BPE exposure group significantly changed compared to the Control group shortly (4 days) and 3 months (90 days) after exposure, both in terms of abundance (weighted UniFrac) and presence only (unweighted UniFrac). Alterations in beta diversity linked to disease state have also been observed in other lung microbiome studies (55). In an *in vivo* inhalation study examining particulate matter from motor vehicles or biomass fuel, similar results to our BPE exposures were also observed—an increase in alpha diversity and shifts in beta diversity (51).

The combination of sand inhalation followed by BPE exposure showed similar results to those observed for BPE exposure alone. The ‘BPE + Sand’ exposure group consistently had microbiome populations similar to those seen in the BPE-only exposure group, indicating that, at least in this exposure scenario, sand did not demonstrate an additive effect in modulating lung microbiome composition. It is possible that the lack of synergistic effects was due to the animal study design, in which the airborne hazards were presented serially rather than as a concurrent exposure set. The lack of synergism is more likely due to the limited influence of sand exposure on the lung microbiome (see section below).

### 4.3 Burn pit emission exposure initiates lasting changes to the lung microbiome

Unexpectedly, a shift in the lung microbiome was observed 4 days post-exposure in BPE-exposed animals. It might be expected that by 90 days post-exposure, the lung microbiome would revert to baseline populations (56). Instead, BPE exposure created alterations to the lung microbiome that were maintained for at least 3 months after exposure, as evidenced by the populations seen in the Burn and ‘Burn + Sand’ exposure groups (Figure 1). Once perturbed, the lung microbiome appeared to normalize to a new microbiome state. It would be of interest to determine whether these changes are longer-lasting or permanent. In one of the few longitudinal studies on the lung microbiome, Woo et al. (57) observed lung microbiome stability in non-cystic fibrosis bronchiectasis patients with an average of 5.8 years between sampling.

At 90-day post-exposure, the genera most associated with the changes in BPE-exposed groups were *Comamonas*, *Pseudomonas*, and *Novosphingobium.* All three genera were also identified as contributing to the statistical difference between the BPE vs. Control group at 4-day post-exposure, in addition to 46 other genera. The genera most associated with the differences at 4 days post-exposure seen between the BPE vs. Control and BPE + Sand vs. Control groups were *Corynebacterium*, *Geobacillus*, *Sphingomonas*, and *Streptococcus*. Although *Sphingomonas* has been linked to reagent contamination (58), all samples in this study were extracted simultaneously using the same kits and subsequently sequenced in the same run. It is also possible that exposure to BPE provides inoculation of the selected species. For example, *Novosphingobium* is a diverse microbe that can degrade aromatic compounds (59) and could have been present in the burned mass and potentially in the emission plume (60, 61).

### 4.4 Sand inhalation not a driving factor in lung microbiome alterations

Four weeks of sand inhalation did not seem to alter lung microbiome populations, as the Sand and Control groups were not significantly different based on alpha or beta diversity, with both groups sharing the most abundant genera. In addition, comparison of Sand vs. Control groups indicated few ASVs responsible for the difference in communities identified at either 4-day or 90-day post-exposure, much fewer than the ASVs seen from control comparisons of the BPE and the ‘BPE + Sand’ groups. The amplitude of differential abundance (log2 FC) was also lower in Sand vs. Control than in BPE vs. Control or ‘BPE + Sand’ vs. Control at both time points. It is known that environmental particulate matter does drive a host immune response (62–65), and Iraqi sand has been shown to induce *in vivo* lung inflammation with fibrosis (66). Therefore, it was not surprising to observe a noticeable population change in animals exposed to sand.

### 4.5 Transition potential for toxicological monitoring, modeling, and risk susceptibility

The lung microbiome comprises a commensal community of specific microorganisms—including bacteria, fungi, and viruses—that inhabit the respiratory tract. Unlike the gut, the healthy lung environment is a low-biomass ecosystem, meaning that it has a relatively sparse microbial population due to constant elimination mechanisms, such as coughing and mucociliary clearance (67). Research on the lung microbiome is in its infancy compared to the much more extensively studied gut microbiome. The intestinal tract is nutrient-rich and hosts the vast majority of the body’s microbial biomass and genetic diversity, compared to the lung’s limited microbiota composition. However, this knowledge gap is narrowing as advanced, non-culture-dependent techniques have confirmed that the lung is not sterile and have revealed the critical role of individualized lung microbiome populations in health outcomes. In addition, recent efforts have revealed the mechanisms of the gut-lung axis, where changes in the gut microbiota could affect lung health and disease (68).

The lung microbiome is emerging as a new tool in inhalation toxicology, as it may mediate the host’s response to environmental exposures. Similar to the gut microbiome, individual lung microbiome populations could impart additional risk or protect against inhaled toxicants. To study how individual lung microbiota populations affect adverse outcomes, specific lung microbiome profile populations could be incorporated into *in vitro* and *in vivo* models to better represent human complexity. Lung microbiome dysbiosis could also serve as a highly sensitive biomarker to identify exposure type and possibly dosage (69). Characterizing the mechanisms of the lung microbiome in modifying the host response would aid in understanding individual variability and susceptibility to inhaled toxicants or materials (70). This information is crucial for improving risk assessment by understanding how unique individual microbial communities influence host metabolism, inflammation, and overall health outcomes following exposure.

### 4.6 Study limitations

Laboratory animals have been shown to have variable lung microbiomes that can be influenced by different shipments, vendors, and cage locations (71). Our study used the same vendor and shipment for all animals used; however, the animals were housed in individual cages, albeit in randomized rack locations. A more significant limitation, especially regarding our Sand exposure data, is that the “4-day” Sand group was actually necropsied 13 days after the final sand exposure day (Figure 1) due to logistical limitations. This could have contributed to the observed lack of significance in the Sand vs. Control results, as the delay may have allowed for a longer post-exposure recovery period to return to baseline LMB populations. However, as our BPE data indicated, if the microbiome was significantly perturbed by sand exposure, it would be unlikely that the LMB would resolve to baseline during the 13-day delay. The study design did not allow for longitudinal or pre-exposure analyses of the same animal due to the sampling methodology (lavage collected after euthanasia). Finally, only male animals were included in this study; it is unknown whether the same responses are observed in female animals. There are indications that inhaled toxicants can induce sex-specific alterations in lung microbiome populations (72).

## 5 Conclusion

This study examined the impact of sub-chronic exposure to burn pit emissions and/or sand on the lungs and lung microbiome using whole-body inhalation exposures. To the best of our knowledge, this study is the first to characterize lung microbiome alterations through a well-controlled *in vivo* approach using an appropriate exposure method (whole-body inhalation) and well-defined exposure components, specifically utilizing actual burn pit emission plumes for BPE and sand collected from a theater near Baghdad, Iraq. Unlike other studies using sputum, lung lavage was collected after euthanasia, generating lung microbiome data that were minimally contaminated by oral or oropharyngeal populations.

While significant shifts in the lung microbiome were observed with BPE exposures, no major inflammatory or debilitating respiratory phenotype was observed in any of the exposure groups (37, 38). This may be an indication that the lung microbiome was directly affected by inhalation of the BPE components rather than a change in the localized environment driven by lung disease or injury, as seen in COPD. With the caveats described above, burn pit emissions appear to be the primary driver of lung microbiome alterations, whereas the impact of sand on lung microbe populations is far less significant. This finding contrasts with the study by Hu et al. (73) which involved exposed mice to PM2.5 (SRM 1649b; Urban Dust; National Institute of Standards and Technology) through intratracheal installation, which exhibited a strong dose-dependent impact on alpha diversity. The Hu study observed an increased abundance of *Bacteroidetes*, *Cyanobacteria*, and *Firmicutes* with PM2.5 exposures.

A longer chronic study period might have resulted in substantial lung damage and potentially greater alterations in the lung microbiome. Despite these limitations, our study data are still useful in examining early lung microbiome alterations in a pre- or early disease state. We are currently examining whether the alterations we observed in several genera might be involved in modulating host gene expression and inflammation. Taken together, the lung microbiome signatures in response to BPE and sand may help to more accurately gauge, monitor, and mitigate health risks associated with uncontrolled burn emissions, dense urban air pollution, and dust events in order to improve operational risk management and in-field and post-deployment medical treatment.

## Data Availability

Singe-end sequences from this project and associated metadata were deposited in the NCBI Sequence Read Archive (PioProject accession ID: PRNJA1391818).

## Author disclaimer

The views expressed in this study are those of the authors and do not reflect the official policy or position of the United States Air Force, Department of Defense, Department of Veteran Affairs, or the United States Government. This material is declared a work of the United States Government and is not subject to copyright protection in the United States of America. This paper has been approved as DISTRIBUTION A: Approved for public release; distribution unlimited. AFIMSC MSC-2021-0049/AFRL 2021-0524 cleared 23 Mar 2021.

## Glossary

ABT: ambient breeze tunnel
AFRL: Air Force Research Laboratory
ASV: amplicon sequence variant
BALF: bronchoalveolar lavage fluid
CO: carbon monoxide
CO_2_: carbon dioxide
COPD: chronic obstructive pulmonary disease
DoD: Department of Defense
gDNA: genomic deoxyribonucleic acid
IOM: Institute of Medicine
LMB: lung microbiome
MSW: mixed solid waste
NAMRU-D: Naval Medical Research Unit Dayton
NTHi: *Haemophilus influenzae* nontypeable strain
PAH: polyaromatic hydrocarbon
PAHs: polyaromatic hydrocarbons
PBS: phosphate-buffered saline
PM: particulate matter
SO_2_: sulfur dioxide
SWA: Southwest Asia
USACEHR: U.S. Army Center of Environmental Health Research
U.S.: United States
VOC: volatile organic acid
