# Supplementary material for: In vivo lung microbiome alterations from burn pit emissions and/or sand inhalation exposures
Source: Front Public Health. 2026 Apr 20;13:1693310. doi: 10.3389/fpubh.2025.1693310 (PMC13137057; doi:10.3389/fpubh.2025.1693310)
Supplement: Supplementary file 1 [file Data_Sheet_1.pdf]

SUPPLEMENTAL INFORMATION

SI Table 1. ASVs Contributing to Change in Microbiome Community Over Time. Analyzed through pairwise comparison for each exposure category to exposures days 4 to 90 in DESeq2 at genus taxonomic level. ASVs removed that were also present in control group analysis. Significance level set at 0.01 before Benjamini-Hochberg correction was applied to obtain an adjusted significance level.

DISTRIBUTION A. Approved for public release; distribution unlimited. Case No. RH-21-121743 cleared 23 Mar 2021.

ASV	Sample	baseMean	log2FoldChange	lfcSE	stat	padj	Phylum	Class	Order	Family	Genus
[illegible]	Bare	[illegible]	[illegible]	[illegible]	[illegible]	[illegible]	p__Bacteroidetes	c__Bacteroidia	o__Bacteroidales	f__[Paraprevotellaceae]	g__[Prevotella]
[illegible]	Bare_Sand	[illegible]	[illegible]	[illegible]	[illegible]	[illegible]	p__Firmicutes	c__Clostridia	o__Clostridiales	f__[Tissierellaceae]	g__1-68
[illegible]	Bare_Sand	[illegible]	[illegible]	[illegible]	[illegible]	[illegible]	p__Firmicutes	c__Clostridia	o__Clostridiales	f__[Tissierellaceae]	g__1-68
[illegible]	Bare_Sand	[illegible]	[illegible]	[illegible]	[illegible]	[illegible]	p__Firmicutes	c__Clostridia	o__Clostridiales	f__[Tissierellaceae]	g__1-68
[illegible]	Bare_Sand	[illegible]	[illegible]	[illegible]	[illegible]	[illegible]	p__Firmicutes	c__Clostridia	o__Clostridiales	f__[Tissierellaceae]	g__1-68
[illegible]	Bare	[illegible]	[illegible]	[illegible]	[illegible]	[illegible]	p__Proteobacteria	c__Gammaproteobacteria	o__Pseudomonadales	f__Moraxellaceae	g__Acinetobacter
[illegible]	Bare	[illegible]	[illegible]	[illegible]	[illegible]	[illegible]	p__Proteobacteria	c__Gammaproteobacteria	o__Pseudomonadales	f__Moraxellaceae	g__Acinetobacter
[illegible]	Bare_Sand	[illegible]	[illegible]	[illegible]	[illegible]	[illegible]	p__Actinobacteria	c__Actinobacteria	o__Actinomycetales	f__Actinomycetaceae	g__Actinobaculum
[illegible]	Bare_Sand	[illegible]	[illegible]	[illegible]	[illegible]	[illegible]	p__Actinobacteria	c__Actinobacteria	o__Actinomycetales	f__Actinomycetaceae	g__Actinobaculum
[illegible]	Control	[illegible]	[illegible]	[illegible]	[illegible]	[illegible]	p__Firmicutes	c__Bacilli	o__Lactobacillales	f__Aerococcaceae	g__Aerococcus
[illegible]	Bare	[illegible]	[illegible]	[illegible]	[illegible]	[illegible]	p__Proteobacteria	c__Alphaproteobacteria	o__Rhizobiales	f__Bradyrhizobiaceae	g__Afipia
[illegible]	Bare	[illegible]	[illegible]	[illegible]	[illegible]	[illegible]	p__Firmicutes	c__Bacilli	o__Lactobacillales	f__Aerococcaceae	g__Alloiococcus
[illegible]	Bare	[illegible]	[illegible]	[illegible]	[illegible]	[illegible]	p__Firmicutes	c__Clostridia	o__Clostridiales	f__[Tissierellaceae]	g__Anaerococcus
[illegible]	Bare	[illegible]	[illegible]	[illegible]	[illegible]	[illegible]	p__Firmicutes	c__Clostridia	o__Clostridiales	f__[Tissierellaceae]	g__Anaerococcus
[illegible]	Bare	[illegible]	[illegible]	[illegible]	[illegible]	[illegible]	p__Bacteroidetes	c__Bacteroidia	o__Bacteroidales	f__Bacteroidaceae	g__Bacteroides
[illegible]	Bare	[illegible]	[illegible]	[illegible]	[illegible]	[illegible]	p__Bacteroidetes	c__Bacteroidia	o__Bacteroidales	f__Bacteroidaceae	g__Bacteroides
[illegible]	Bare	[illegible]	[illegible]	[illegible]	[illegible]	[illegible]	p__Bacteroidetes	c__Bacteroidia	o__Bacteroidales	f__Bacteroidaceae	g__Bacteroides
[illegible]	Bare	[illegible]	[illegible]	[illegible]	[illegible]	[illegible]	p__Bacteroidetes	c__Bacteroidia	o__Bacteroidales	f__Bacteroidaceae	g__Bacteroides
[illegible]	Bare	[illegible]	[illegible]	[illegible]	[illegible]	[illegible]	p__Bacteroidetes	c__Bacteroidia	o__Bacteroidales	f__Bacteroidaceae	g__Bacteroides
[illegible]	Bare	[illegible]	[illegible]	[illegible]	[illegible]	[illegible]	p__Proteobacteria	c__Alphaproteobacteria	o__Rhizobiales	f__Bradyrhizobiaceae	g__Balneimonas
[illegible]	Bare	[illegible]	[illegible]	[illegible]	[illegible]	[illegible]	p__Firmicutes	c__Clostridia	o__Clostridiales	f__Lachnospiraceae	g__Blautia
[illegible]	Bare	[illegible]	[illegible]	[illegible]	[illegible]	[illegible]	p__Firmicutes	c__Clostridia	o__Clostridiales	f__Lachnospiraceae	g__Blautia
[illegible]	Bare	[illegible]	[illegible]	[illegible]	[illegible]	[illegible]	p__Proteobacteria	c__Alphaproteobacteria	o__Rhizobiales	f__Bradyrhizobiaceae	g__Bradyrhizobium
[illegible]	Bare_Sand	[illegible]	[illegible]	[illegible]	[illegible]	[illegible]	p__Proteobacteria	c__Alphaproteobacteria	o__Rhizobiales	f__Bradyrhizobiaceae	g__Bradyrhizobium
[illegible]	Bare_Sand	[illegible]	[illegible]	[illegible]	[illegible]	[illegible]	p__Proteobacteria	c__Alphaproteobacteria	o__Rhizobiales	f__Bradyrhizobiaceae	g__Bradyrhizobium
[illegible]	Bare_Sand	[illegible]	[illegible]	[illegible]	[illegible]	[illegible]	p__Proteobacteria	c__Alphaproteobacteria	o__Rhizobiales	f__Bradyrhizobiaceae	g__Bradyrhizobium
[illegible]	Bare_Sand	[illegible]	[illegible]	[illegible]	[illegible]	[illegible]	p__Bacteroidetes	c__Flavobacteriia	o__Flavobacteriales	f__[Weeksellaceae]	g__Chryseobacterium
[illegible]	Bare	[illegible]	[illegible]	[illegible]	[illegible]	[illegible]	p__Firmicutes	c__Clostridia	o__Clostridiales	f__Clostridiaceae	g__Clostridium
[illegible]	Bare	[illegible]	[illegible]	[illegible]	[illegible]	[illegible]	p__Actinobacteria	c__Actinobacteria	o__Actinomycetales	f__Corynebacteriaceae	g__Corynebacterium
[illegible]	Bare	[illegible]	[illegible]	[illegible]	[illegible]	[illegible]	p__Actinobacteria	c__Actinobacteria	o__Actinomycetales	f__Corynebacteriaceae	g__Corynebacterium
[illegible]	Bare	[illegible]	[illegible]	[illegible]	[illegible]	[illegible]	p__Actinobacteria	c__Actinobacteria	o__Actinomycetales	f__Corynebacteriaceae	g__Corynebacterium
[illegible]	Bare	[illegible]	[illegible]	[illegible]	[illegible]	[illegible]	p__Actinobacteria	c__Actinobacteria	o__Actinomycetales	f__Corynebacteriaceae	g__Corynebacterium
[illegible]	Bare	[illegible]	[illegible]	[illegible]	[illegible]	[illegible]	p__Actinobacteria	c__Actinobacteria	o__Actinomycetales	f__Corynebacteriaceae	g__Corynebacterium
[illegible]	Bare	[illegible]	[illegible]	[illegible]	[illegible]	[illegible]	p__Actinobacteria	c__Actinobacteria	o__Actinomycetales	f__Corynebacteriaceae	g__Corynebacterium
[illegible]	Bare	[illegible]	[illegible]	[illegible]	[illegible]	[illegible]	p__Actinobacteria	c__Actinobacteria	o__Actinomycetales	f__Corynebacteriaceae	g__Corynebacterium
[illegible]	Bare_Sand	[illegible]	[illegible]	[illegible]	[illegible]	[illegible]	p__Actinobacteria	c__Actinobacteria	o__Actinomycetales	f__Corynebacteriaceae	g__Corynebacterium
[illegible]	Sand	[illegible]	[illegible]	[illegible]	[illegible]	[illegible]	p__Actinobacteria	c__Actinobacteria	o__Actinomycetales	f__Corynebacteriaceae	g__Corynebacterium
[illegible]	Bare	[illegible]	[illegible]	[illegible]	[illegible]	[illegible]	p__[Thermi]	c__Deinococci	o__Deinococcales	f__Deinococcaceae	g__Deinococcus
[illegible]	Bare	[illegible]	[illegible]	[illegible]	[illegible]	[illegible]	p__[Thermi]	c__Deinococci	o__Deinococcales	f__Deinococcaceae	g__Deinococcus
[illegible]	Bare	[illegible]	[illegible]	[illegible]	[illegible]	[illegible]	p__[Thermi]	c__Deinococci	o__Deinococcales	f__Deinococcaceae	g__Deinococcus
[illegible]	Bare	[illegible]	[illegible]	[illegible]	[illegible]	[illegible]	p__Proteobacteria	c__Betaproteobacteria	o__Burkholderiales	f__Comamonadaceae	g__Delftia
[illegible]	Bare_Sand	[illegible]	[illegible]	[illegible]	[illegible]	[illegible]	p__Bacteroidetes	c__Cytophagia	o__Cytophagales	f__Cytophagaceae	g__Dyadobacter
[illegible]	Bare_Sand	[illegible]	[illegible]	[illegible]	[illegible]	[illegible]	p__Bacteroidetes	c__Cytophagia	o__Cytophagales	f__Cytophagaceae	g__Dyadobacter
[illegible]	Bare	[illegible]	[illegible]	[illegible]	[illegible]	[illegible]	p__Proteobacteria	c__Gammaproteobacteria	o__Pseudomonadales	f__Moraxellaceae	g__Enhydrobacter
[illegible]	Bare	[illegible]	[illegible]	[illegible]	[illegible]	[illegible]	p__Bacteroidetes	c__Flavobacteriia	o__Flavobacteriales	f__Flavobacteriaceae	g__Flavobacterium
[illegible]	Control	[illegible]	[illegible]	[illegible]	[illegible]	[illegible]	p__Bacteroidetes	c__Flavobacteriia	o__Flavobacteriales	f__Flavobacteriaceae	g__Flavobacterium
[illegible]	Bare_Sand	[illegible]	[illegible]	[illegible]	[illegible]	[illegible]	p__Actinobacteria	c__Actinobacteria	o__Actinomycetales	f__Nocardioidaceae	g__Friedmanniella
[illegible]	Bare_Sand	[illegible]	[illegible]	[illegible]	[illegible]	[illegible]	p__Actinobacteria	c__Actinobacteria	o__Actinomycetales	f__Nocardioidaceae	g__Friedmanniella
[illegible]	Bare	[illegible]	[illegible]	[illegible]	[illegible]	[illegible]	p__Firmicutes	c__Bacilli	o__Bacillales	f__Bacillaceae	g__Geobacillus
[illegible]	Bare	[illegible]	[illegible]	[illegible]	[illegible]	[illegible]	p__Firmicutes	c__Bacilli	o__Bacillales	f__Bacillaceae	g__Geobacillus
[illegible]	Bare	[illegible]	[illegible]	[illegible]	[illegible]	[illegible]	p__Firmicutes	c__Bacilli	o__Bacillales	f__Bacillaceae	g__Geobacillus
[illegible]	Bare	[illegible]	[illegible]	[illegible]	[illegible]	[illegible]	p__Firmicutes	c__Bacilli	o__Bacillales	f__Bacillaceae	g__Geobacillus
[illegible]	Bare	[illegible]	[illegible]	[illegible]	[illegible]	[illegible]	p__Firmicutes	c__Bacilli	o__Bacillales	f__Bacillaceae	g__Geobacillus
[illegible]	Bare	[illegible]	[illegible]	[illegible]	[illegible]	[illegible]	p__Proteobacteria	c__Gammaproteobacteria	o__Pasteurellales	f__Pasteurellaceae	g__Haemophilus
[illegible]	Bare	[illegible]	[illegible]	[illegible]	[illegible]	[illegible]	p__Proteobacteria	c__Alphaproteobacteria	o__Rhizobiales	f__Hyphomicrobiaceae	g__Hyphomicrobium
[illegible]	Bare_Sand	[illegible]	[illegible]	[illegible]	[illegible]	[illegible]	p__Proteobacteria	c__Alphaproteobacteria	o__Sphingomonadales	f__Sphingomonadaceae	g__Kaistobacter
[illegible]	Bare_Sand	[illegible]	[illegible]	[illegible]	[illegible]	[illegible]	p__Proteobacteria	c__Alphaproteobacteria	o__Sphingomonadales	f__Sphingomonadaceae	g__Kaistobacter
[illegible]	Bare	[illegible]	[illegible]	[illegible]	[illegible]	[illegible]	p__Actinobacteria	c__Actinobacteria	o__Actinomycetales	f__Micrococcaceae	g__Kocuria
[illegible]	Bare	[illegible]	[illegible]	[illegible]	[illegible]	[illegible]	p__Firmicutes	c__Bacilli	o__Lactobacillales	f__Lactobacillaceae	g__Lactobacillus
[illegible]	Bare	[illegible]	[illegible]	[illegible]	[illegible]	[illegible]	p__Firmicutes	c__Bacilli	o__Lactobacillales	f__Lactobacillaceae	g__Lactobacillus
[illegible]	Sand	[illegible]	[illegible]	[illegible]	[illegible]	[illegible]	p__Firmicutes	c__Bacilli	o__Lactobacillales	f__Lactobacillaceae	g__Lactobacillus
[illegible]	Sand	[illegible]	[illegible]	[illegible]	[illegible]	[illegible]	p__Firmicutes	c__Bacilli	o__Lactobacillales	f__Lactobacillaceae	g__Lactobacillus
[illegible]	Sand	[illegible]	[illegible]	[illegible]	[illegible]	[illegible]	p__Firmicutes	c__Bacilli	o__Lactobacillales	f__Lactobacillaceae	g__Lactobacillus
[illegible]	Bare_Sand	[illegible]	[illegible]	[illegible]	[illegible]	[illegible]	p__Firmicutes	c__Bacilli	o__Lactobacillales	f__Streptococcaceae	g__Lactococcus
[illegible]	Bare_Sand	[illegible]	[illegible]	[illegible]	[illegible]	[illegible]	p__Firmicutes	c__Bacilli	o__Lactobacillales	f__Streptococcaceae	g__Lactococcus
[illegible]	Bare	[illegible]	[illegible]	[illegible]	[illegible]	[illegible]	p__Proteobacteria	c__Gammaproteobacteria	o__Xanthomonadales	f__Xanthomonadaceae	g__Luteimonas
[illegible]	Bare	[illegible]	[illegible]	[illegible]	[illegible]	[illegible]	p__Proteobacteria	c__Alphaproteobacteria	o__Rhizobiales	f__Methylobacteriaceae	g__Methylobacterium
[illegible]	Bare	[illegible]	[illegible]	[illegible]	[illegible]	[illegible]	p__Proteobacteria	c__Alphaproteobacteria	o__Rhizobiales	f__Methylobacteriaceae	g__Methylobacterium
[illegible]	Bare_Sand	[illegible]	[illegible]	[illegible]	[illegible]	[illegible]	p__Proteobacteria	c__Alphaproteobacteria	o__Rhizobiales	f__Methylobacteriaceae	g__Methylobacterium
[illegible]	Bare_Sand	[illegible]	[illegible]	[illegible]	[illegible]	[illegible]	p__Proteobacteria	c__Alphaproteobacteria	o__Rhizobiales	f__Methylobacteriaceae	g__Methylobacterium
[illegible]	Bare	[illegible]	[illegible]	[illegible]	[illegible]	[illegible]	p__Actinobacteria	c__Actinobacteria	o__Actinomycetales	f__Micrococcaceae	g__Micrococcus
[illegible]	Bare	[illegible]	[illegible]	[illegible]	[illegible]	[illegible]	p__Actinobacteria	c__Actinobacteria	o__Actinomycetales	f__Micrococcaceae	g__Micrococcus
[illegible]	Sand	[illegible]	[illegible]	[illegible]	[illegible]	[illegible]	p__Actinobacteria	c__Actinobacteria	o__Actinomycetales	f__Micrococcaceae	g__Micrococcus
[illegible]	Bare	[illegible]	[illegible]	[illegible]	[illegible]	[illegible]	p__Actinobacteria	c__Actinobacteria	o__Actinomycetales	f__Mycobacteriaceae	g__Mycobacterium
[illegible]	Bare_Sand	[illegible]	[illegible]	[illegible]	[illegible]	[illegible]	p__Actinobacteria	c__Actinobacteria	o__Actinomycetales	f__Mycobacteriaceae	g__Mycobacterium
[illegible]	Bare_Sand	[illegible]	[illegible]	[illegible]	[illegible]	[illegible]	p__Actinobacteria	c__Actinobacteria	o__Actinomycetales	f__Mycobacteriaceae	g__Mycobacterium
[illegible]	Bare_Sand	[illegible]	[illegible]	[illegible]	[illegible]	[illegible]	p__Actinobacteria	c__Actinobacteria	o__Actinomycetales	f__Mycobacteriaceae	g__Mycobacterium
[illegible]	Bare_Sand	[illegible]	[illegible]	[illegible]	[illegible]	[illegible]	p__Actinobacteria	c__Actinobacteria	o__Actinomycetales	f__Mycobacteriaceae	g__Mycobacterium
[illegible]	Bare_Sand	[illegible]	[illegible]	[illegible]	[illegible]	[illegible]	p__Actinobacteria	c__Actinobacteria	o__Actinomycetales	f__Mycobacteriaceae	g__Mycobacterium
[illegible]	Bare_Sand	[illegible]	[illegible]	[illegible]	[illegible]	[illegible]	p__Actinobacteria	c__Actinobacteria	o__Actinomycetales	f__Mycobacteriaceae	g__Mycobacterium
[illegible]	Bare_Sand	[illegible]	[illegible]	[illegible]	[illegible]	[illegible]	p__Actinobacteria	c__Actinobacteria	o__Actinomycetales	f__Mycobacteriaceae	g__Mycobacterium
[illegible]	Bare	[illegible]	[illegible]	[illegible]	[illegible]	[illegible]	p__Proteobacteria	c__Gammaproteobacteria	o__Xanthomonadales	f__Sinobacteraceae	g__Nevskia
[illegible]	Bare_Sand	[illegible]	[illegible]	[illegible]	[illegible]	[illegible]	p__Actinobacteria	c__Actinobacteria	o__Actinomycetales	f__Nocardioidaceae	g__Nocardioides
[illegible]	Bare	[illegible]	[illegible]	[illegible]	[illegible]	[illegible]	p__Proteobacteria	c__Alphaproteobacteria	o__Sphingomonadales	f__Sphingomonadaceae	g__Novosphingobium
[illegible]	Bare	[illegible]	[illegible]	[illegible]	[illegible]	[illegible]	p__Proteobacteria	c__Alphaproteobacteria	o__Sphingomonadales	f__Sphingomonadaceae	g__Novosphingobium
[illegible]	Bare	[illegible]	[illegible]	[illegible]	[illegible]	[illegible]	p__Proteobacteria	c__Alphaproteobacteria	o__Rhizobiales	f__Brucellaceae	g__Ochrobactrum
[illegible]	Bare	[illegible]	[illegible]	[illegible]	[illegible]	[illegible]	p__Bacteroidetes	c__Bacteroidia	o__Bacteroidales	f__Porphyromonadaceae	g__Parabacteroides
[illegible]	Bare_Sand	[illegible]	[illegible]	[illegible]	[illegible]	[illegible]	p__Bacteroidetes	c__Sphingobacteriia	o__Sphingobacteriales	f__Sphingobacteriaceae	g__Pedobacter
[illegible]	Bare_Sand	[illegible]	[illegible]	[illegible]	[illegible]	[illegible]	p__Firmicutes	c__Clostridia	o__Clostridiales	f__[Tissierellaceae]	g__Peptoniphilus
[illegible]	Bare_Sand	[illegible]	[illegible]	[illegible]	[illegible]	[illegible]	p__Firmicutes	c__Clostridia	o__Clostridiales	f__[Tissierellaceae]	g__Peptoniphilus
[illegible]	Bare_Sand	[illegible]	[illegible]	[illegible]	[illegible]	[illegible]	p__Firmicutes	c__Clostridia	o__Clostridiales	f__[Tissierellaceae]	g__Peptoniphilus
[illegible]	Bare_Sand	[illegible]	[illegible]	[illegible]	[illegible]	[illegible]	p__Firmicutes	c__Clostridia	o__Clostridiales	f__[Tissierellaceae]	g__Peptoniphilus
[illegible]	Bare_Sand	[illegible]	[illegible]	[illegible]	[illegible]	[illegible]	p__Firmicutes	c__Clostridia	o__Clostridiales	f__[Tissierellaceae]	g__Peptoniphilus
[illegible]	Bare_Sand	[illegible]	[illegible]	[illegible]	[illegible]	[illegible]	p__Firmicutes	c__Clostridia	o__Clostridiales	f__[Tissierellaceae]	g__Peptoniphilus
[illegible]	Bare_Sand	[illegible]	[illegible]	[illegible]	[illegible]	[illegible]	p__Bacteroidetes	c__Bacteroidia	o__Bacteroidales	f__Porphyromonadaceae	g__Porphyromonas
[illegible]	Bare	[illegible]	[illegible]	[illegible]	[illegible]	[illegible]	p__Bacteroidetes	c__Bacteroidia	o__Bacteroidales	f__Prevotellaceae	g__Prevotella
[illegible]	Bare_Sand	[illegible]	[illegible]	[illegible]	[illegible]	[illegible]	p__Bacteroidetes	c__Bacteroidia	o__Bacteroidales	f__Prevotellaceae	g__Prevotella
[illegible]	Bare_Sand	[illegible]	[illegible]	[illegible]	[illegible]	[illegible]	p__Bacteroidetes	c__Bacteroidia	o__Bacteroidales	f__Prevotellaceae	g__Prevotella
[illegible]	Bare	[illegible]	[illegible]	[illegible]	[illegible]	[illegible]	p__Actinobacteria	c__Actinobacteria	o__Actinomycetales	f__Propionibacteriaceae	g__Propionibacterium
[illegible]	Bare	[illegible]	[illegible]	[illegible]	[illegible]	[illegible]	p__Actinobacteria	c__Actinobacteria	o__Actinomycetales	f__Propionibacteriaceae	g__Propionibacterium
[illegible]	Bare	[illegible]	[illegible]	[illegible]	[illegible]	[illegible]	p__Proteobacteria	c__Gammaproteobacteria	o__Enterobacteriales	f__Enterobacteriaceae	g__Proteus
[illegible]	Control	[illegible]	[illegible]	[illegible]	[illegible]	[illegible]	p__Proteobacteria	c__Gammaproteobacteria	o__Enterobacteriales	f__Enterobacteriaceae	g__Proteus
[illegible]	Bare	[illegible]	[illegible]	[illegible]	[illegible]	[illegible]	p__Proteobacteria	c__Gammaproteobacteria	o__Pseudomonadales	f__Pseudomonadaceae	g__Pseudomonas
[illegible]	Bare	[illegible]	[illegible]	[illegible]	[illegible]	[illegible]	p__Proteobacteria	c__Gammaproteobacteria	o__Pseudomonadales	f__Pseudomonadaceae	g__Pseudomonas
[illegible]	Bare	[illegible]	[illegible]	[illegible]	[illegible]	[illegible]	p__Proteobacteria	c__Gammaproteobacteria	o__Pseudomonadales	f__Pseudomonadaceae	g__Pseudomonas
[illegible]	Bare	[illegible]	[illegible]	[illegible]	[illegible]	[illegible]	p__Proteobacteria	c__Gammaproteobacteria	o__Pseudomonadales	f__Pseudomonadaceae	g__Pseudomonas
[illegible]	Bare	[illegible]	[illegible]	[illegible]	[illegible]	[illegible]	p__Proteobacteria	c__Gammaproteobacteria	o__Pseudomonadales	f__Pseudomonadaceae	g__Pseudomonas
[illegible]	Bare	[illegible]	[illegible]	[illegible]	[illegible]	[illegible]	p__Proteobacteria	c__Gammaproteobacteria	o__Pseudomonadales	f__Pseudomonadaceae	g__Pseudomonas
[illegible]	Bare	[illegible]	[illegible]	[illegible]	[illegible]	[illegible]	p__Proteobacteria	c__Gammaproteobacteria	o__Pseudomonadales	f__Pseudomonadaceae	g__Pseudomonas
[illegible]	Bare	[illegible]	[illegible]	[illegible]	[illegible]	[illegible]	p__Proteobacteria	c__Gammaproteobacteria	o__Pseudomonadales	f__Pseudomonadaceae	g__Pseudomonas
[illegible]	Bare	[illegible]	[illegible]	[illegible]	[illegible]	[illegible]	p__Proteobacteria	c__Gammaproteobacteria	o__Pseudomonadales	f__Pseudomonadaceae	g__Pseudomonas
[illegible]	Control	[illegible]	[illegible]	[illegible]	[illegible]	[illegible]	p__Proteobacteria	c__Gammaproteobacteria	o__Pseudomonadales	f__Pseudomonadaceae	g__Pseudomonas
[illegible]	Sand	[illegible]	[illegible]	[illegible]	[illegible]	[illegible]	p__Proteobacteria	c__Gammaproteobacteria	o__Pseudomonadales	f__Pseudomonadaceae	g__Pseudomonas
[illegible]	Sand	[illegible]	[illegible]	[illegible]	[illegible]	[illegible]	p__Proteobacteria	c__Gammaproteobacteria	o__Pseudomonadales	f__Pseudomonadaceae	g__Pseudomonas
[illegible]	Bare	[illegible]	[illegible]	[illegible]	[illegible]	[illegible]	p__Firmicutes	c__Clostridia	o__Clostridiales	f__Peptococcaceae	g__rc4-4
[illegible]	Bare	[illegible]	[illegible]	[illegible]	[illegible]	[illegible]	p__Proteobacteria	c__Alphaproteobacteria	o__Rhodospirillales	f__Acetobacteraceae	g__Roseomonas
[illegible]	Bare	[illegible]	[illegible]	[illegible]	[illegible]	[illegible]	p__Proteobacteria	c__Alphaproteobacteria	o__Rhodospirillales	f__Acetobacteraceae	g__Roseomonas
[illegible]	Sand	[illegible]	[illegible]	[illegible]	[illegible]	[illegible]	p__Actinobacteria	c__Actinobacteria	o__Actinomycetales	f__Micrococcaceae	g__Rothia
[illegible]	Bare	[illegible]	[illegible]	[illegible]	[illegible]	[illegible]	p__Actinobacteria	c__Rubrobacteria	o__Rubrobacterales	f__Rubrobacteraceae	g__Rubrobacter
[illegible]	Bare	[illegible]	[illegible]	[illegible]	[illegible]	[illegible]	p__Firmicutes	c__Clostridia	o__Clostridiales	f__Ruminococcaceae	g__Ruminococcus
[illegible]	Bare	[illegible]	[illegible]	[illegible]	[illegible]	[illegible]	p__Firmicutes	c__Clostridia	o__Clostridiales	f__Ruminococcaceae	g__Ruminococcus
[illegible]	Bare	[illegible]	[illegible]	[illegible]	[illegible]	[illegible]	p__Firmicutes	c__Clostridia	o__Clostridiales	f__Ruminococcaceae	g__Ruminococcus
[illegible]	Control	[illegible]	[illegible]	[illegible]	[illegible]	[illegible]	p__Proteobacteria	c__Gammaproteobacteria	o__Enterobacteriales	f__Enterobacteriaceae	g__Salmonella
[illegible]	Control	[illegible]	[illegible]	[illegible]	[illegible]	[illegible]	p__Proteobacteria	c__Gammaproteobacteria	o__Enterobacteriales	f__Enterobacteriaceae	g__Salmonella
[illegible]	Control	[illegible]	[illegible]	[illegible]	[illegible]	[illegible]	p__Proteobacteria	c__Gammaproteobacteria	o__Enterobacteriales	f__Enterobacteriaceae	g__Salmonella
[illegible]	Control	[illegible]	[illegible]	[illegible]	[illegible]	[illegible]	p__Proteobacteria	c__Gammaproteobacteria	o__Enterobacteriales	f__Enterobacteriaceae	g__Salmonella
[illegible]	Control	[illegible]	[illegible]	[illegible]	[illegible]	[illegible]	p__Proteobacteria	c__Gammaproteobacteria	o__Enterobacteriales	f__Enterobacteriaceae	g__Salmonella
[illegible]	Bare	[illegible]	[illegible]	[illegible]	[illegible]	[illegible]	p__Firmicutes	c__Bacilli	o__Bacillales	f__Planococcaceae	g__Solibacillus
[illegible]	Bare	[illegible]	[illegible]	[illegible]	[illegible]	[illegible]	p__Firmicutes	c__Bacilli	o__Bacillales	f__Planococcaceae	g__Solibacillus
[illegible]	Control	[illegible]	[illegible]	[illegible]	[illegible]	[illegible]	p__Firmicutes	c__Bacilli	o__Bacillales	f__Planococcaceae	g__Solibacillus
[illegible]	Bare_Sand	[illegible]	[illegible]	[illegible]	[illegible]	[illegible]	p__Proteobacteria	c__Alphaproteobacteria	o__Sphingomonadales	f__Sphingomonadaceae	g__Sphingobium
[illegible]	Bare_Sand	[illegible]	[illegible]	[illegible]	[illegible]	[illegible]	p__Proteobacteria	c__Alphaproteobacteria	o__Sphingomonadales	f__Sphingomonadaceae	g__Sphingobium
[illegible]	Bare_Sand	[illegible]	[illegible]	[illegible]	[illegible]	[illegible]	p__Proteobacteria	c__Alphaproteobacteria	o__Sphingomonadales	f__Sphingomonadaceae	g__Sphingobium
[illegible]	Bare	[illegible]	[illegible]	[illegible]	[illegible]	[illegible]	p__Proteobacteria	c__Alphaproteobacteria	o__Sphingomonadales	f__Sphingomonadaceae	g__Sphingomonas
[illegible]	Bare	[illegible]	[illegible]	[illegible]	[illegible]	[illegible]	p__Proteobacteria	c__Alphaproteobacteria	o__Sphingomonadales	f__Sphingomonadaceae	g__Sphingomonas
[illegible]	Bare	[illegible]	[illegible]	[illegible]	[illegible]	[illegible]	p__Proteobacteria	c__Alphaproteobacteria	o__Sphingomonadales	f__Sphingomonadaceae	g__Sphingomonas
[illegible]	Bare	[illegible]	[illegible]	[illegible]	[illegible]	[illegible]	p__Proteobacteria	c__Alphaproteobacteria	o__Sphingomonadales	f__Sphingomonadaceae	g__Sphingomonas
[illegible]	Bare	[illegible]	[illegible]	[illegible]	[illegible]	[illegible]	p__Proteobacteria	c__Alphaproteobacteria	o__Sphingomonadales	f__Sphingomonadaceae	g__Sphingomonas
[illegible]	Bare	[illegible]	[illegible]	[illegible]	[illegible]	[illegible]	p__Proteobacteria	c__Alphaproteobacteria	o__Sphingomonadales	f__Sphingomonadaceae	g__Sphingomonas
[illegible]	Bare	[illegible]	[illegible]	[illegible]	[illegible]	[illegible]	p__Proteobacteria	c__Alphaproteobacteria	o__Sphingomonadales	f__Sphingomonadaceae	g__Sphingomonas
[illegible]	Bare	[illegible]	[illegible]	[illegible]	[illegible]	[illegible]	p__Proteobacteria	c__Alphaproteobacteria	o__Sphingomonadales	f__Sphingomonadaceae	g__Sphingomonas
[illegible]	Bare	[illegible]	[illegible]	[illegible]	[illegible]	[illegible]	p__Proteobacteria	c__Alphaproteobacteria	o__Sphingomonadales	f__Sphingomonadaceae	g__Sphingomonas
[illegible]	Bare	[illegible]	[illegible]	[illegible]	[illegible]	[illegible]	p__Proteobacteria	c__Alphaproteobacteria	o__Sphingomonadales	f__Sphingomonadaceae	g__Sphingomonas
[illegible]	Bare	[illegible]	[illegible]	[illegible]	[illegible]	[illegible]	p__Proteobacteria	c__Alphaproteobacteria	o__Sphingomonadales	f__Sphingomonadaceae	g__Sphingomonas
[illegible]	Bare	[illegible]	[illegible]	[illegible]	[illegible]	[illegible]	p__Proteobacteria	c__Alphaproteobacteria	o__Sphingomonadales	f__Sphingomonadaceae	g__Sphingomonas
[illegible]	Bare	[illegible]	[illegible]	[illegible]	[illegible]	[illegible]	p__Firmicutes	c__Bacilli	o__Bacillales	f__Staphylococcaceae	g__Staphylococcus
[illegible]	Bare	[illegible]	[illegible]	[illegible]	[illegible]	[illegible]	p__Firmicutes	c__Bacilli	o__Bacillales	f__Staphylococcaceae	g__Staphylococcus
[illegible]	Bare	[illegible]	[illegible]	[illegible]	[illegible]	[illegible]	p__Firmicutes	c__Bacilli	o__Bacillales	f__Staphylococcaceae	g__Staphylococcus
[illegible]	Bare	[illegible]	[illegible]	[illegible]	[illegible]	[illegible]	p__Firmicutes	c__Bacilli	o__Bacillales	f__Staphylococcaceae	g__Staphylococcus
[illegible]	Bare	[illegible]	[illegible]	[illegible]	[illegible]	[illegible]	p__Firmicutes	c__Bacilli	o__Bacillales	f__Staphylococcaceae	g__Staphylococcus
[illegible]	Bare	[illegible]	[illegible]	[illegible]	[illegible]	[illegible]	p__Firmicutes	c__Bacilli	o__Bacillales	f__Staphylococcaceae	g__Staphylococcus
[illegible]	Bare	[illegible]	[illegible]	[illegible]	[illegible]	[illegible]	p__Firmicutes	c__Bacilli	o__Bacillales	f__Staphylococcaceae	g__Staphylococcus
[illegible]	Bare_Sand	[illegible]	[illegible]	[illegible]	[illegible]	[illegible]	p__Firmicutes	c__Bacilli	o__Bacillales	f__Staphylococcaceae	g__Staphylococcus
[illegible]	Bare_Sand	[illegible]	[illegible]	[illegible]	[illegible]	[illegible]	p__Firmicutes	c__Bacilli	o__Bacillales	f__Staphylococcaceae	g__Staphylococcus
[illegible]	Bare_Sand	[illegible]	[illegible]	[illegible]	[illegible]	[illegible]	p__Firmicutes	c__Bacilli	o__Bacillales	f__Staphylococcaceae	g__Staphylococcus
[illegible]	Bare_Sand	[illegible]	[illegible]	[illegible]	[illegible]	[illegible]	p__Firmicutes	c__Bacilli	o__Bacillales	f__Staphylococcaceae	g__Staphylococcus
[illegible]	Bare_Sand	[illegible]	[illegible]	[illegible]	[illegible]	[illegible]	p__Firmicutes	c__Bacilli	o__Bacillales	f__Staphylococcaceae	g__Staphylococcus
[illegible]	Bare_Sand	[illegible]	[illegible]	[illegible]	[illegible]	[illegible]	p__Firmicutes	c__Bacilli	o__Bacillales	f__Staphylococcaceae	g__Staphylococcus
[illegible]	Sand	[illegible]	[illegible]	[illegible]	[illegible]	[illegible]	p__Firmicutes	c__Bacilli	o__Bacillales	f__Staphylococcaceae	g__Staphylococcus
[illegible]	Bare	[illegible]	[illegible]	[illegible]	[illegible]	[illegible]	p__Proteobacteria	c__Gammaproteobacteria	o__Xanthomonadales	f__Xanthomonadaceae	g__Stenotrophomonas
[illegible]	Bare	[illegible]	[illegible]	[illegible]	[illegible]	[illegible]	p__Proteobacteria	c__Gammaproteobacteria	o__Xanthomonadales	f__Xanthomonadaceae	g__Stenotrophomonas
[illegible]	Bare	[illegible]	[illegible]	[illegible]	[illegible]	[illegible]	p__Firmicutes	c__Bacilli	o__Lactobacillales	f__Streptococcaceae	g__Streptococcus
[illegible]	Bare_Sand	[illegible]	[illegible]	[illegible]	[illegible]	[illegible]	p__Firmicutes	c__Bacilli	o__Lactobacillales	f__Streptococcaceae	g__Streptococcus
[illegible]	Bare_Sand	[illegible]	[illegible]	[illegible]	[illegible]	[illegible]	p__Firmicutes	c__Bacilli	o__Lactobacillales	f__Streptococcaceae	g__Streptococcus
[illegible]	Control	[illegible]	[illegible]	[illegible]	[illegible]	[illegible]	p__Firmicutes	c__Bacilli	o__Lactobacillales	f__Streptococcaceae	g__Streptococcus
[illegible]	Control	[illegible]	[illegible]	[illegible]	[illegible]	[illegible]	p__Firmicutes	c__Bacilli	o__Lactobacillales	f__Streptococcaceae	g__Streptococcus
[illegible]	Bare	[illegible]	[illegible]	[illegible]	[illegible]	[illegible]	p__Proteobacteria	c__Betaproteobacteria	o__Burkholderiales	f__Alcaligenaceae	g__Sutterella
[illegible]	Bare	[illegible]	[illegible]	[illegible]	[illegible]	[illegible]	p__Proteobacteria	c__Betaproteobacteria	o__Burkholderiales	f__Alcaligenaceae	g__Sutterella
[illegible]	Control	[illegible]	[illegible]	[illegible]	[illegible]	[illegible]	p__Firmicutes	c__Bacilli	o__Lactobacillales	f__Enterococcaceae	g__Vagococcus
[illegible]	Bare_Sand	[illegible]	[illegible]	[illegible]	[illegible]	[illegible]	p__Firmicutes	c__Clostridia	o__Clostridiales	f__[Tissierellaceae]	g__WAL_1855D

DISTRIBUTION A. Approved for public release; distribution unlimited. Case No. RH-21-121743 cleared 23 Mar 2021.

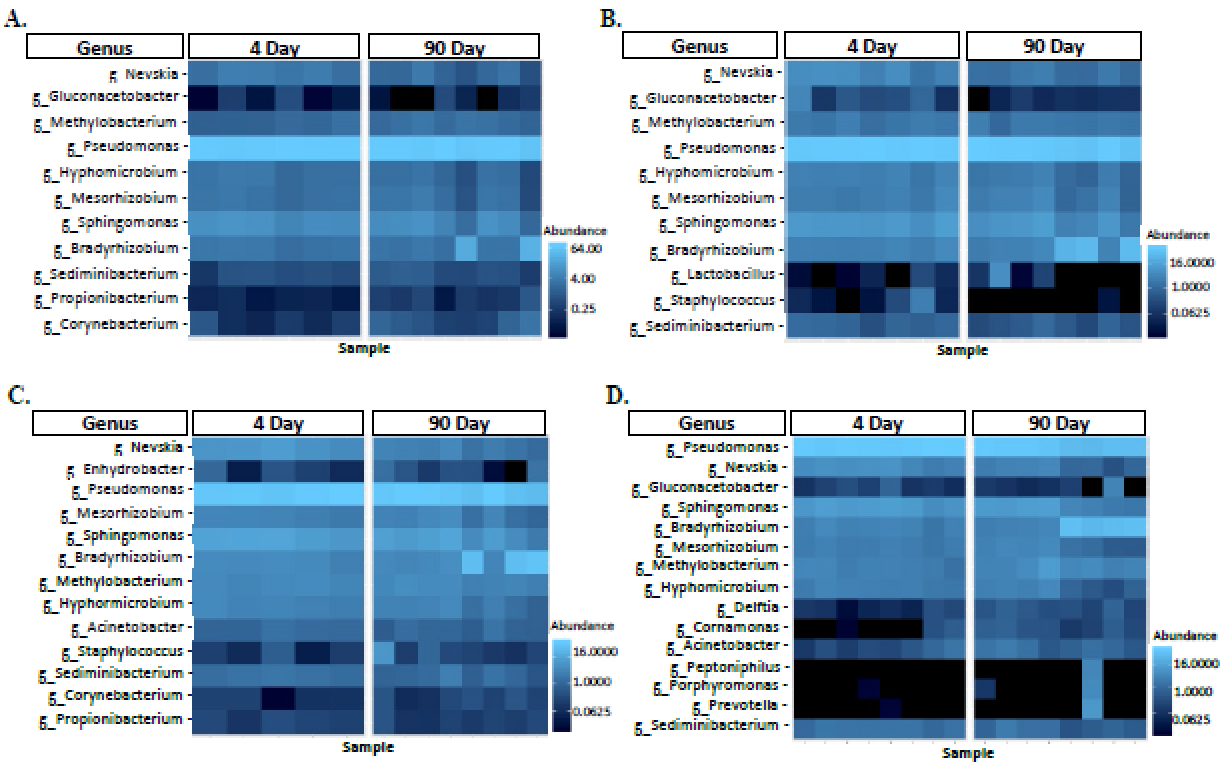

SI Figure 1. Top 100 most abundant ASVs, separated by sample type of (A) Control, (B) Sand, (C) BPE, and (D) BPE + Sand.

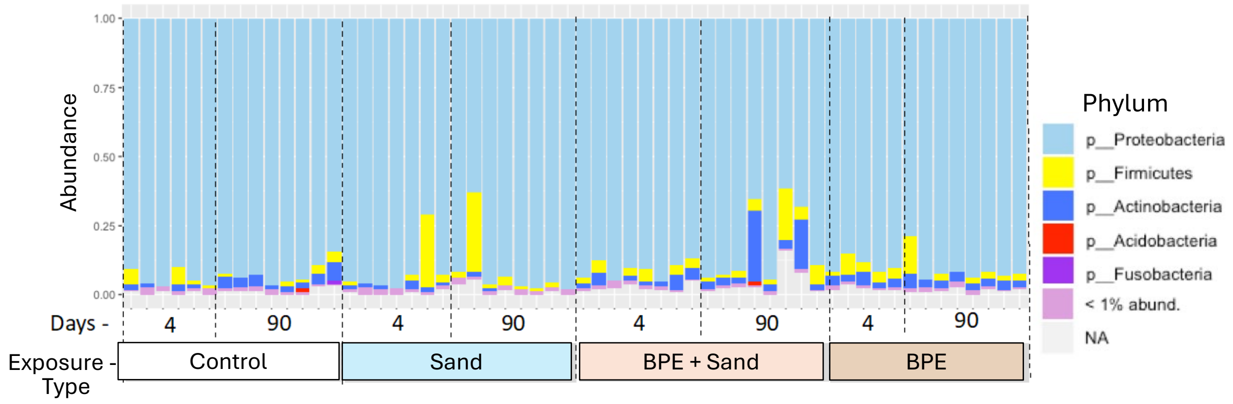

SI Figure 2. 4-day and 90-day post-exposure lung Microbiome by exposure type, at taxonomic Phylum level. Relative abundance at taxonomic class, showing only classes with average of 5% or more relative abundance.

DISTRIBUTION A. Approved for public release; distribution unlimited. Case No. RH-21-121743 cleared 23 Mar 2021.

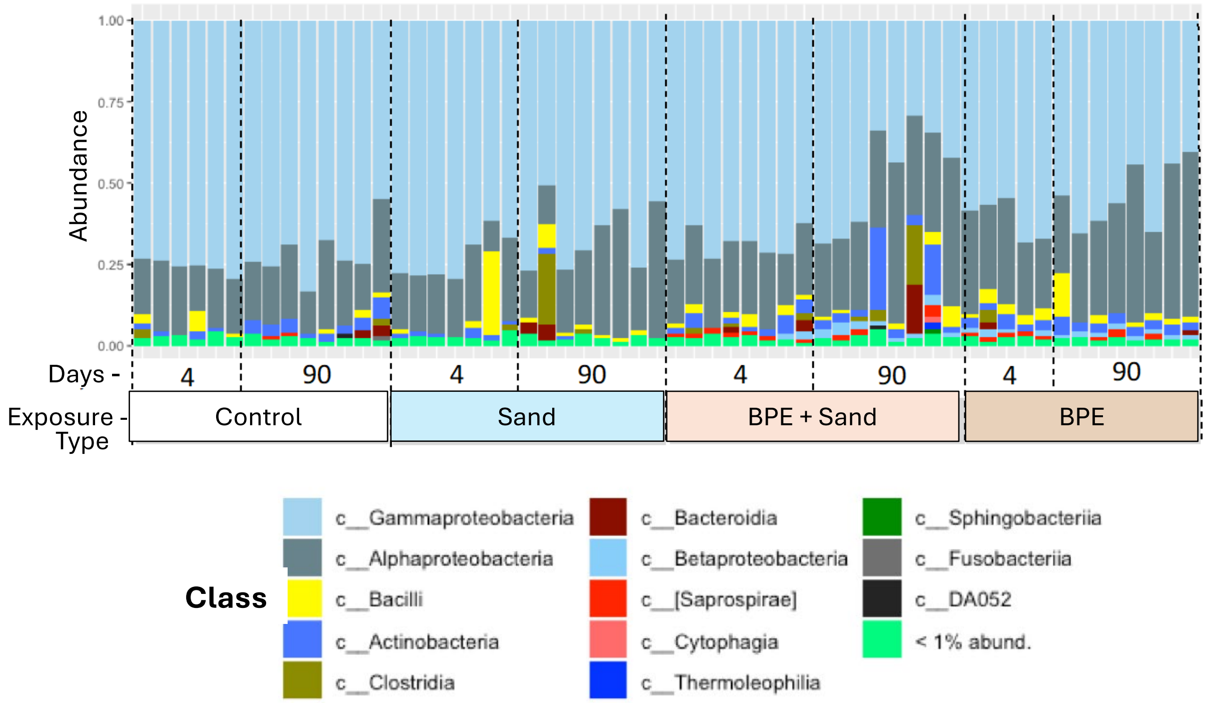

SI Figure 3. 4-day and 90-day post-exposure lung Microbiome by exposure type, at taxonomic Class level. Relative abundance at taxonomic class, showing only classes with average of 5% or more relative abundance.

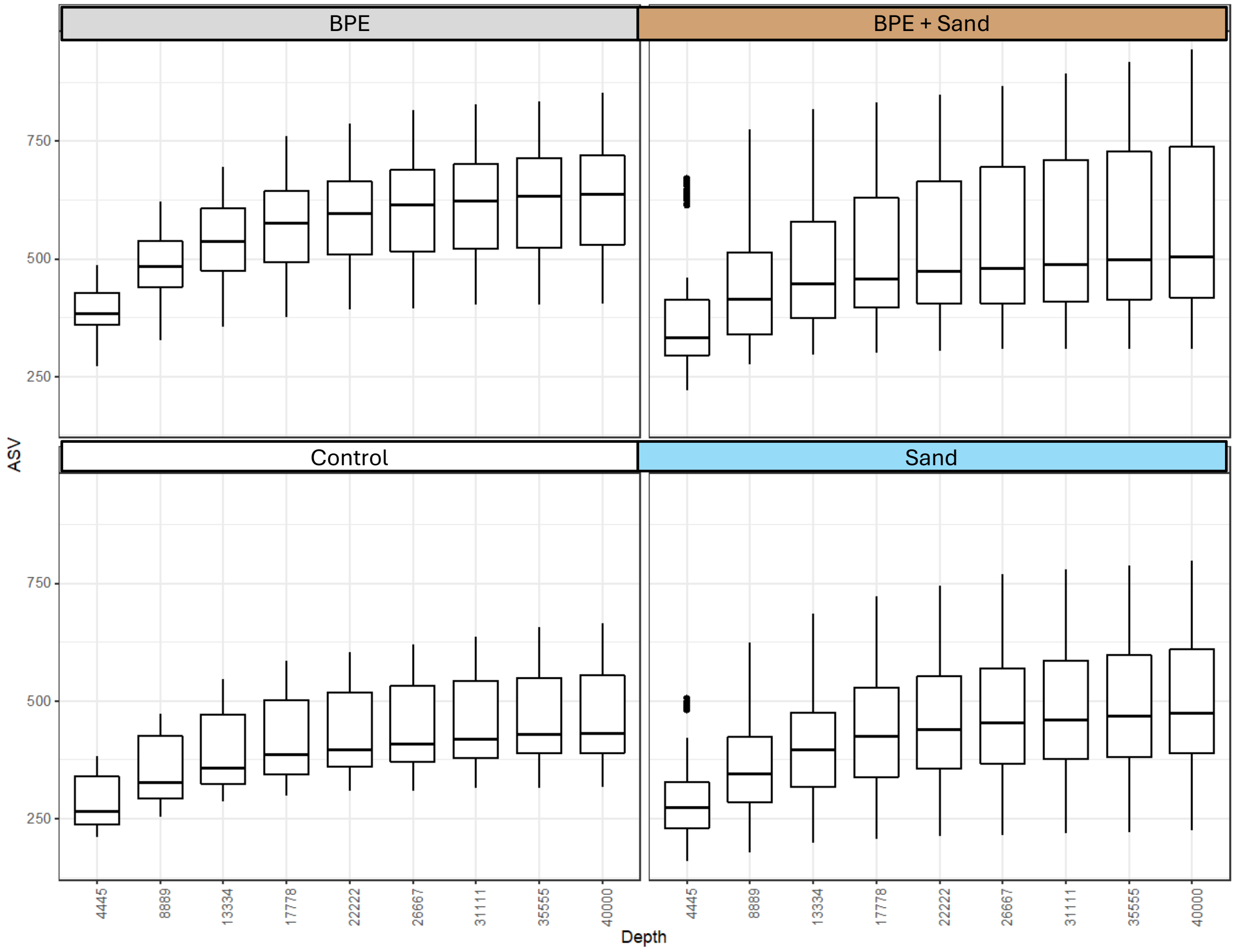

SI Figure 4. Observed ASVs based on exposure.

DISTRIBUTION A. Approved for public release; distribution unlimited. Case No. RH-21-121743 cleared 23 Mar 2021.

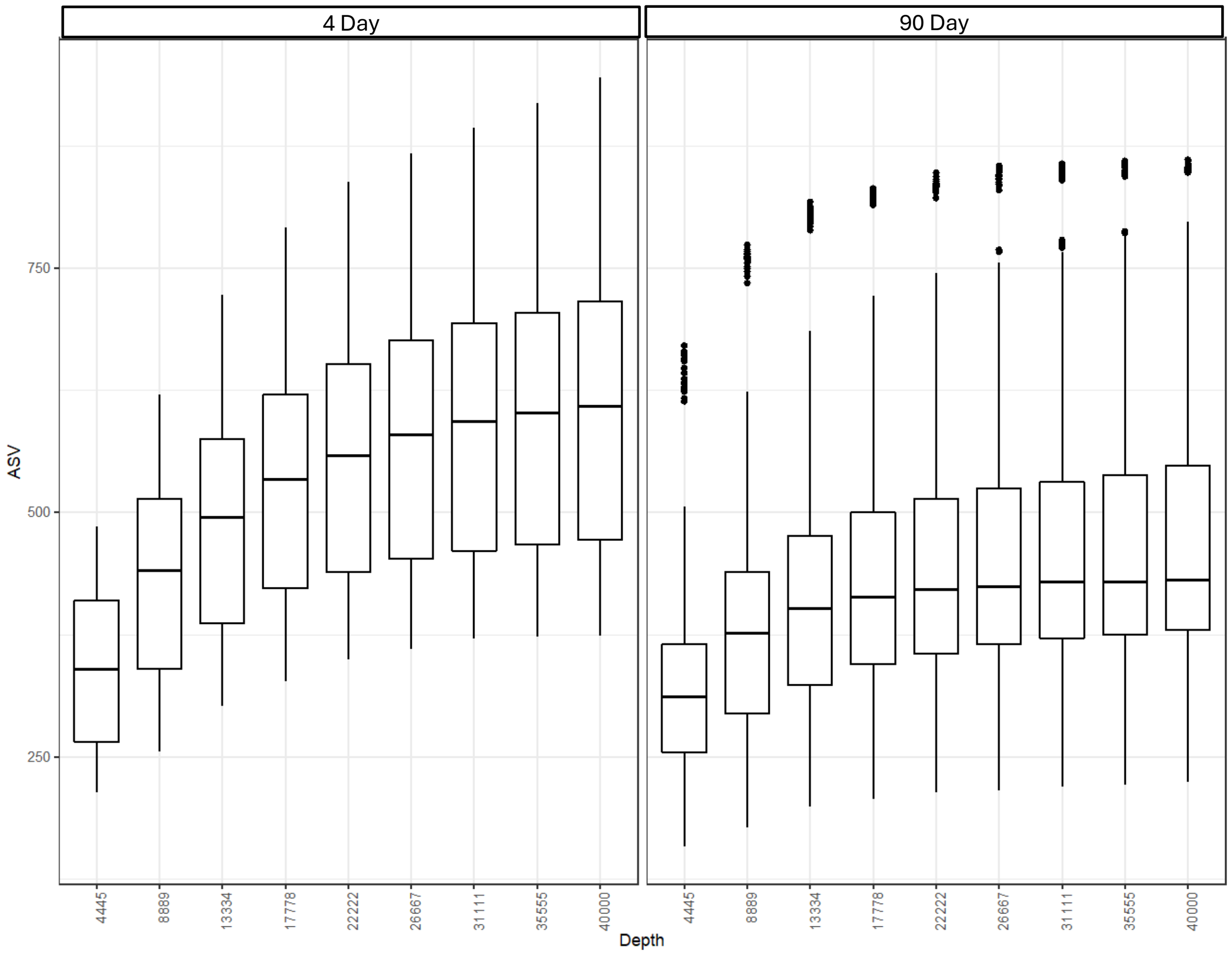

SI Figure 5. Observed ASVs based on day.
